# Effectiveness of deep brain stimulation on refractory aggression in pediatric patients with autism and severe intellectual disability: meta-analytic review

**DOI:** 10.1186/s12887-024-04920-x

**Published:** 2024-07-30

**Authors:** Jorge Herrera-Pino, Juancarlos Benedetti-Isaac, Daniela Ripoll-Córdoba, Loida Camargo, Edgard E. Castillo-Tamara, Breiner Morales-Asencio, Esther Perea-Castro, Martín Torres Zambrano, Alejandro Ducassou, Yuliana Flórez, María F. Porto, Pascual A. Gargiulo, Boris Zurita-Cueva, Nicole Caldichoury, Juan-Carlos Coronado, Cesar Castellanos, Cleto Ramírez-Penso, Norman López

**Affiliations:** 1 College of Medicine, Florida International University, 11200 SW 8Th St, Miami, FL 33199 USA; 2 Clinica Neurocardiovascular, Neurodinamia, Tv. 54 #21a-75, Cartagena, Colombia; 3 Misericordia International Clinic, Cra. 74 #76-105, Barranquilla, 080001 Colombia; 4 Departamento de Ciencias Sociales, Universidad de La Costa, Cl. 58 #55 - 66, Barranquilla, 080002 Colombia; 5 Facultad de Medicina, Universidad de Cartagena, Campus Zaragocilla, Cartagena de Indias, Bolívar 130014 Colombia; 6 Facultad de Medicina, Universidad del Sinú, Provincia de Cartagena, Calle 30 #20-71, Cartagena de Indias, Bolívar 130001 Colombia; 7 Universidad Mayor, Av. Alemania 281, Temuco, Araucanía, 4801043 Chile; 8 Department of Cognition, Development and Educational Psychology, Universitat de Barcelona and Bellvitge Institute for Biomedical Research (IDIBELL), Carrer de La Feixa Llarga, L’Hospitalet de Llobregat, Barcelona 08907 Spain; 9 Laboratorio de Neurociencias y Psicología Experimental (CONICET), Departamento de Patología, Facultad de Ciencias Médicas, Universidad Nacional de Cuyo. Parque General San Martín, Mendoza, M5502JMA Argentina; 10 Departamento de Neurocirugía, Omni Hospital, Avenida abel Romeo Castillo y ave. Tanca Marengo., Guayaquil, 090513 Ecuador; 11 Departamento de Ciencias Sociales, Universidad de Los Lagos, Av Alberto-Hertha Fuchslocher 1305, Osorno, Los Lagos, Chile; 12 Facultad de Salud, Universidad Católica de Temuco, Montt 56, Temuco, Araucanía 4780000 Chile; 13 Instituto Dominicano para el Estudio de la Salud Integral y la Psicología Aplicada (IDESIP), C. Eugenio Deschamps No.5, Santo Domingo, 10014 República Dominicana; 14 Departamento de Neurocirugía, Director general del Centro Cardio-Neuro-Oftalmológico y Trasplante (CECANOT), C/ Federico Velázquez #1, Sector Maria Auxiliadora, Santo Domingo, República Dominicana; 15 Sociedad Dominicana de Neurología y Neurocirugía (Pax- President), F38M+CHM, Santo Domingo, 10106 República Dominicana; 16 Escuela de Kinesiología, Facultad de Salud, Universidad Santo Tomás, Manuel Rodríguez 060, Temuco, 4790870 Chile

**Keywords:** Deep brain stimulation, Autism, Intellectual disability, Aggressive, Metanalysis

## Abstract

Some patients with autism and severe intellectual disability may experience uncontrolled aggression, causing serious injury or harm to others, and the therapeutic ineffectiveness of traditional pharmacological and behavioral treatment may aggravate symptoms. Deep brain stimulation (DBS) has been tested in patients with little evidence in children and adolescents. Therefore, we analyzed the efficacy and safety of DBS in refractory aggression in pediatric subjects with autism (ASD) and severe intelligence deficit (ID).

**Methods** A meta-analytic review of Web of Science (WOS) and Scopus articles, following Prisma criteria. A total of 555 articles were identified, but after applying the inclusion criteria, only 18 were analyzed. The review of the registries and the extraction of information was performed by 2 independent groups, to reduce the evaluator’s bias. For the description of the results, pediatric patients with ASD or ID present in each registry, with an application of specialized scales (Overt aggression scale, OAS, and THE modified version of the OAS, MOAS) pre and post-DBS, with a clinical follow-up of at least 12 months, were considered valid. Clinical improvement was calculated using tests of aggressiveness. In each registry with available data and then pooling the means of all patients in the OAS and MOAS, the effect size of DBS (overall and per study) was estimated. Finally, the adapted NOS scale was applied to rate the studies’ quality and level of bias.

**Results** In the studies analyzed, 65/100 were pediatric patients, with a mean age of 16.8 years. Most of the studies were conducted in South America and Europe. In all teams, aggressive behavior was intractable, but only 9 groups (53/65) applied specialized scales to measure aggressiveness, and of these, only 51 subjects had a follow-up of at least 12 months. Thus, in 48/51 a clinical improvement of patients was estimated (94.2%), with a considerable overall effect size (OAS: *d* = 4.32; MOAS: *d* = 1.46). However, adverse effects and complications were found in 13/65 subjects undergoing DBS. The brain target with the most evidence and the fewest side effects was the posteromedial hypothalamic nuclei (pHypN). Finally, applying the adapted NOS scale, quality, and bias, only 9 studies show the best indicators.

**Conclusion** An optimal level of efficacy was found in only half of the publications. This is mainly due to design errors and irrelevant information in the reports. We believe that DBS in intractable aggressiveness in children and adolescents with ASD and severe ID can be safe and effective if working groups apply rigorous criteria for patient selection, interdisciplinary assessments, objective scales for aggressiveness, and known surgical targets.

**Supplementary Information:**

The online version contains supplementary material available at 10.1186/s12887-024-04920-x.

## Introduction

Aggressive, self-injurious and uncontrolled behavior usually occurs in a subset of pediatric patients with autism (ASD) and severe intellectual disability (SID). Dysfunctional behavior and aggressive and maladaptive responses are typical in these subjects [1, 2]. Severe forms of ASD and ID put the safety and well-being of patients at risk, creating a challenge for the family and caregivers [3]. As a result, these subjects are subjected to direct mechanical coercive restraint measures, using protective helmets to avoid craniofacial fractures, gloves, and restraint belts to avoid self-mutilation and injuries to third parties [3–5]. Moreover, the consequences of highly aggressive behaviors are aggravated by the ineffectiveness and refractoriness of symptoms to pharmacological and psychological treatment [6]. The main medications to manage or reduce persistent aggression usually include antipsychotic drugs, with or without benzodiazepines, mood stabilizers, antiepileptics, alpha-2 agonists, beta-blockers, lithium, and selective serotonin reuptake inhibitors, among others. Unfortunately, despite the combination of drugs and doses used, due to the severity of the clinical symptoms, some of these patients remain refractory to treatment [7, 8]. Unfortunately, despite the combination of drugs and doses used, the severity of clinical symptoms forces to classify these patients as untreatable [7].

For this subset of patients with uncontrolled aggression, neurosurgical interventions have been developed [9]. Deep brain stimulation (DBS) involves the implantation of electrodes in specific brain regions, where a neurostimulator applies electrical impulses to treat neurological and psychiatric pathologies refractory to conventional treatment. DBS is an effective, reversible, and safe treatment for a wide variety of intractable clinical conditions [7, 10, 11]. It is especially effective for patients with dangerous, self-injurious or third-party injurious behaviors resistant to traditional treatment [6, 9, 12–14].

Due to the frustration and refractoriness of pharmacological and behavioral treatments, DBS is gaining particular interest among specialists and family members [15, 16]. Unfortunately, the evidence for the application of DBS in children and adolescents is progressing slowly [17]. Therefore, the aims of this study were: 1) to summarize the current knowledge on the effectiveness and safety of DBS for aggressive and intractable behavior in pediatric patients with ASD and SID; 2) to analyze the technical aspects of DBS, and 3) to estimate the quality levels of available studies.

## Method

The Prisma guidelines [18] were followed for the meta-analytic review. The search strategy consisted of an exhaustive literature review by consulting WOS (Web of Science) and Scopus databases on July 28, 2023; using the following search criteria, which were limited to human studies, in English and Spanish: SCOPUS: ( ALL ( ALL (“Deep brain stimulation”) OR ALL (dbs) AND ALL (aggressiv*) AND ALL (autism) OR ALL (“Autism Spectrum Disorder”) OR ALL (“Intellectual disability”)) AND (LIMIT-TO ( DOCTYPE, “ar”) OR LIMIT-TO ( DOCTYPE, “le”)) AND (LIMIT-TO ( LANGUAGE, “English”) OR LIMIT-TO ( LANGUAGE, “Spanish”)). WOS: “Deep brain stimulation” (Topic) OR DBS (Topic) OR Neurosurgery (Topic) AND Autism (Topic) OR “Autism Spectrum Disorder” (Topic) OR “Intellectual disability” (Topic) AND aggressiv* (Topic) OR “aggressive behavior” (Topic) OR “Intractable aggressiveness” (Topic) AND “disruptive behavior” (All Fields) and Article or Letter (Document Types) and English or Spanish (Languages) and “Deep Brain Stimulation” (Search within all fields) and Children (Search within all fields).

### Procedure

A double-masked procedure was applied to select the studies. A first working group (DA, JE, BM, NC) reviewed each article's title, abstract and keywords, applying the following inclusion criteria: patients undergoing DBS with intractable aggression; with Autism or Intellectual Disability; minors (children or adolescents). The American Pediatric Society guidelines were followed, which establish the pediatric population aged 21 years or younger [19, 20]. In contrast, we excluded studies that would apply other neuromodulation techniques, focused on adults (≥ 22 years), that would apply DBS in pediatric populations with other disorders, or that did not intervene in refractory aggressiveness. Then, a second working group (A, B, C, D, E) extracted the information from the selected records and eventual discrepancies were resolved with the principal investigators (JCBI/NL).

### Statistical analysis

For the description of the results, only pediatric subjects included in each selected article were accepted as valid, with objective assessment of aggressiveness and post-surgical follow-up of at least 12 months. Considering that several registries applied DBS to children and adults or did not have disaggregated or complete information, such as test means, standard deviation, and effect size, we performed an exhaustive review of each study. We constructed these statistical values or consulted each study’s principal authors to obtain the missing information. We selected objective and specialized scales assessing aggression (OAS, MOAS, BPAQ) with available data and a pre-post DBS application to calculate the percentage of clinical improvement. Then, Student’s t was applied to each study separately, and Cohen’s d was used to calculate the effect size of the DBS for significant results [21]. The interpretation of Cohen’s d is a small effect (0.15–0.40), a medium effect (0.40–0.75) and a significant effect (+ 0.75). Likewise, a pre-and post-DBS intragroup analysis was performed with a Student’s t-test, grouping the mean scores and the standard deviation obtained by all patients in the aggressiveness tests; this to estimate the overall effect size of the DBS on the aggressiveness of the participants. All analyses were processed with SPSS 25 software.

### Evaluation of study quality and bias

Subsequently, the quality of the studies was analyzed using the adjusted version of the Newcastle–Ottawa Scale (NOS) for non-comparative cohort studies. In meetings with the work team, we adjusted the NOS questions, considering the relevant information that studies of this type should contain [22–25]. The result was 5 questions summarizing the NOS Scale criteria adjusted to studies with DBS. Each item is scored as positive (1) or negative (0). The questions were as follows: 1) Did the sample represent all the patients treated at the medical center, i.e., were all the patients treated in the period studied included in the study (1 point)? 2) Was there a correct diagnosis (ASD/ID) (0.5 points) and adequate identification of the clinical problem? I.e. intractable, drug-resistant, or uncontrolled aggressiveness (0.5 points). 3) Was the postoperative follow-up period equal to or greater than 12 months (1 point). 4) Were all important data cited in the report? That is, was there an adequate clinical evaluation (0.25 points), as well as a relevant psychometric assessment (0.25 points); in addition, were the implantation (0.25 points) and brain stimulation parameters reported (0.25 points)? (0.25 points). 5) Were the results obtained by applying objective and specialized scales for aggression (1 point)?

Finally, we evaluated the bias in the records considering the NOS scale. The evaluation guideline contained 4 dimensions: 1) Selection of participants, which included aspects of representativeness and selection criteria; 2) Comparability, which was based on the review of methodological aspects of the registries, to identify the technical rigor in the procedure of each design; 3) Results, where we reviewed whether the studies reported clinical improvement, objective measures of comparison, surgical parameters, side effects and complications; 4) Adequacy of follow-up, mainly preoperative and postoperative with a minimum of 12 months in both cases (See supplementary data 1: Bias assessment guideline).

## Results

### Results of the bibliographic search

**Fig. 1:**
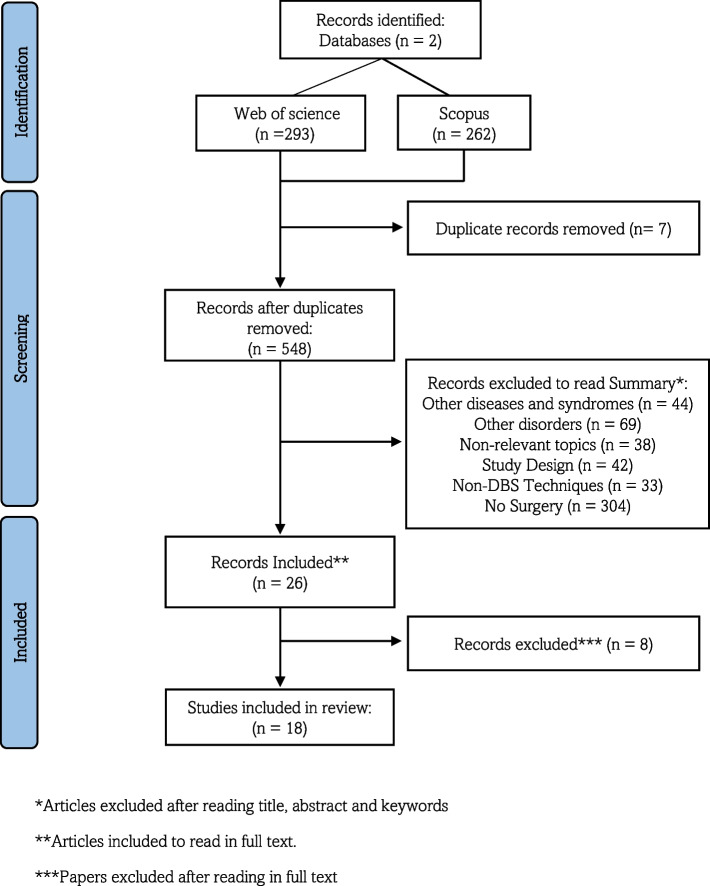
Prism flow chart of the study

The summary of the study selection process can be seen in Fig. 1. The initial search focused on studies in English and Spanish, identifying 555 records (293 from WOS and 262 from Scopus). No studies were obtained from other sources, considering these two databases contain the most relevant global publications. After excluding duplicate records [7], 548 studies were selected for screening. After a review of the title, abstract and keywords, 530 articles were excluded for the following reasons: other diseases or syndromes [26], other disorders [27], not relevant topics [28], other types of study [29], techniques other than DBS [30], patients were not surgically intervened (304). Therefore, 26 studies in English and Spanish were chosen for the complete review. Of these, 8 were excluded, as they did not meet the criteria of age (1), or diagnosis of intellectual disability, ASD, and aggressiveness (2), did not intervene surgically (3), published data from previous studies (1) and did not record cases of aggressiveness (1) (See supplementary data: Excluded studies). Finally, 18 publications met the inclusion criteria and were analyzed.

### Characteristics of the studies

According to Table 1, of the total number of studies selected, 7 were conducted in Colombia; of these, 4 were conducted in a single center located on the northern coast of the country [23–25, 31] and 3 in the central [32–34]. Another 8 registries were conducted in Europe, 2 in the USA and one in Korea. The articles selected, were retrospective studies or case reports, published between 2010 and 2023 in English. In all studies, DBS was applied to 100 subjects, of which 65 corresponded to pediatric patients. Of the latter, 57/65 had intellectual disability (Severe: 23.08%; Moderate: 13.85%), 16/65 had Autism (severe: 10.77%), and 10/65 had mixed comorbidity (Severe: 12.31%). However, the severity of the disorders was not comprehensively reported in all cases [33, 35, 36]. The same situation occurred with the sex of the patients [12, 24, 31, 37], managing to identify only 22 males and 7 females. The average age at the time of surgery was 16.86 years (Range: 10 to 21 years).

**Table 1:** Characteristics of the studies and general information on the participants

Author	Year	Country	Design	Included subjects	Pediatric patients	ASD	Severity ASD	ID	Severity ID	Age, ME, Rank	Sex
Benedetti-Isaac	2015	Colombia	RCS	5	3	1	Severe	3	Severe	2PTE:16; 1PTE:19 (ME:17; R:16–19)	2 M/1F
Benedetti-Isaac	2021	Colombia	RCS	19	19	0	-	19	Severe	(ME: 18,4; R: 14–20)	N/A
Benedetti-Isaac	2023a	Colombia	RCS	5	5	5	Severe	0	-	(ME: 15,2; R: 12–17)	4 M/1F
Benedetti-Isaac	2023b	Colombia	RCS	12	12	0	-	12	Severe	(ME: 15,34; R: 1,80)	N/A
Escobar-Vidarte	2022	Colombia	CR	11	8	1	N/A	8	2Mod/6Sev	(ME: 14,75; R: 10–21)	7 M/1F
Franzini	2013	Italy	RCS	7	2	0	-	2	Severe	(ME: 20,5; R: 20–21)	N/A
Giordano	2016	Italy	CR	1	1	0	-	1	Moderate	21 years	M
Harat	2021	Poland	RCS	6	2	1	Severe	1	Moderate	(ME: 17,5; R:16) PTE1: 16; PTE2: 19	N/A
Heiden	2022	Germany	RCS	10	1	1	Severe	0	-	13 years	M
Kakko	2019	Finland	CR	1	1	1	N/A	1	Moderate	19 years	M
López-Ríos	2022	Colombia	CR	1	1	1	N/A	1	Severe	16 years	F
Maley	2010	USA	CR	1	1	0	-	1	Moderate	19 years	F
Micieli	2017	Colombia	RCS	4	4	0	-	4	3Mod/1Sev	ME:15,25; R:10–19	1F/3 M
Park	2017	Korea	CR	1	1	1	N/A	1	N/A	14	M
Stocco	2014	USA	CR	2	2	2	Severe	2	Severe	(ME: 17,66; R: 17–19) PTE1: 19; PTE2: 17	1 M/1F
Sturm	2012	Germany	CR	1	1	1	Severe	1	Severe	13 years	M
Torres	2013	Spain	RCS	6	1	1	Severe	1	Severe	17 years	M
Torres	2020	Spain	RCS	7							

*RCS* Retrospective case series, *CR* Case report, *ASD* Autism Spectrum Disorders, *ID* Intellectual Disability, *PTE* Patient, *ME* Media, *R* Rank, *M* Male, *F* Female, *N/A* Not available

### Clinical information and follow-up

As shown in Table 2, in 79% of patients (49/65), the etiology of aggressive behavior was defined as “cryptogenic” [12, 23–25, 30–32, 35, 38]. In the remaining participants, congenital diseases were reported [12, 33, 37, 38], or concomitant with perinatal syndromes and events [5, 6, 25, 36, 39–41]. The presence of intractable aggressive, self-aggressive and hetero-aggressive behavior, as well as ASD and ID, was described in all registries. The most frequent comorbidities were medication-resistant abnormal movements [38], psychiatric disorders [35, 37–40], neurodevelopmental syndromes and diseases [6, 30, 32–34, 41] and drug abuse [40]. Although in 4 registries, no comorbidities were reported [23, 24, 31, 39]. The main clinical reason for subjecting patients to DBS was to reduce uncontrolled aggression [5, 6, 12, 23–25, 30–34, 36–41], movement disorder [6, 12, 38] and excessive agitation [35]. Finally, the average clinical follow-up was 42.5 months. In 15 records, follow-up was greater than 12 months; but in 3 studies, it was less than this time [25, 38, 39]; a single study exhibited a follow-up of 163 months [6, 41] and another did not yield any information [35].

**Table 2:** Clinical and follow-up data of the patients

Author	Year	Etiology	Clinical features	Comorbidities	Pre-DBS Treatment	Indication of DBS?	Follow-up in months (Range)
Benedetti-Isaac	2015	1 TS, 2 cryptogenic	IAB, SID, SASD	DRE, Epi	FNA, FBTL, LVTA, OXZA	RUA	PTE1-2: 36 y 48 months; PTE3: 2 months
Benedetti-Isaac	2021	Cryptogenic	IAB, SID	N/A	Pharmacological, psychological	RUA	18 months
Benedetti-Isaac	2023a	Cryptogenic	IAB, SASD	N/A	CNA, VPO, ARZ, Qna, DPM, psychological	RUA	18 months
Benedetti-Isaac	2023b	Cryptogenic	IAB, SID	N/A	BNA y psychological	RUA	18 months
Escobar-Vidarte	2022	Cryptogenic	IAB, SID, ASD	Epi, DS	Pharmacological	RUA	48 months (2-10 years)
Franzini	2013	Cryptogenic	IAB; SID	Epi	CLP, DPM, CHZE, CLNE, BRPM, HPL.	RUA, Epi	PTE 1:60 months; PTE 2: 36 months
Giordano	2016	PH	MID; IAB	PS	LTM, CNA, HPL, SRT, CHZE, CL, VA, RNA, CLP.	RUA, IED	22 months
Harat	2021	Tourette Syndrome	IAB; MID, SASD, SIB, Previous DBS (PTE1)	Vocal and motor tics; Epi, OCD	TPO, PDA, HZA, TNO;	RUA	PTE1: 36 months; PTE2:84 months
Heiden	2022	HIE	SIB, SASD	N/A	Pharmacological	RUA	6 months.
Kakko	2019	Cryptogenic	MID, ASD, IAB, SIB	Epi, TD	VPO, RNA, CLO, HPL; FTA; CLP; DPM; VPO	SIB, TD	N/A
López-Ríos	2022	CdCS	HSCR, ASD, SID, IAB, SIB	Epi, NDD	CNA, RNA, ARZ	RUA	48 monts
Maley	2010	Congenital, BHA	IAB; MID	BD, Depression, DA	CNA, OLZA, kLP, CLP, BNA	RUA	24 months
Micieli	2017	SS, Hypothy, Epi, TS, WS, MTS.	MID, SID, IAB	WS, Epi	DXINA	RUA	27 months
Park	2017	N/A	ASD, ID, SIB	GTC, Impulsivity	AV, TPO, QNA, FNA, psychological	RUA, SIB	24 months
Stoco	2014	1 cromosomic; 1 cryptogenic	SIB, Stereotip, TD	PA, Anxiety	RNA, BNA, TRCS	Stereotypies, RUA	PTE1: 13 months; PTE2: 6 months
Sturm	2012	Cryptogenic	SASD, SID, SIB	KS, ICP	y psychological	RUA	24 months
Torres	2013	SO	SID, SIB, SASD, IAB	Eretism, DRA	TPO, RNA, ARZ, LZPM	Eretism, RUA	163 months (13.5 years)
	2020				GBPA, VPO, LTM, OLZA		

Etiology. *TS* Tuberous Sclerosis, *HIE* Hypoxic-Ischemic Encephalopathy, *HSCR* Hirschsprung’s Disease, *BHA* Bilateral Hippocampal Atrophy, *SS* Sotos Syndrome, *Hypothy* Hypothyroidism, *Epi* Epilepsy, *WS* West Syndrome, *MTS* Mesial Temporal Sclerosis so: Stereotatic operation, *PH* perinatal hypoxia, *N/A* Not available. Clinical Features. *IAB* Intractable Aggressive Behavior, *SID* Severe Intellectual Disability, *SASD* Severe Autism Spectrum Disorder, *MID* Moderate Intellectual Disability, *SIB* Self-injurious Behavior, *ASD* Autism Spectrum Disorder, *ID* Intellectual Disability, *CdCS* Cri du Chat syndrome, *PS* Psychomotor retardation. Comorbidities. *DRE* Drug-Resistant Epilepsy, *DS* Dravet Syndrome, *OCD* Obsessive Compulsive Disorder, *TD* Tardive Dyskinesia, *NDD* Neurodevelopmental Delay, *BD* Bipolar Disorder, *DA* Drug Abuse, *GTC* Generalized Tonic–Clonic, *PA* Progressive Arthritis, *KS* Kanner Syndrome, *ICP* Infantile Cerebral Palsy, *DRA* Drug-Resistant Aggressiveness, *AX* Anxiety. Indication of DBS. *RUA* Reduce Uncontrolled Aggressiveness, *TD* Tardive Dyskinesia, *IED* Intermittent explosive disease. Pharmacological treatment: *CNA* Carbamazepine, *VA* Valproic acid, *DTO* Divalproate, *QNA* Quetiapine, *RNA* Risperidone, *BRPM* Bromazepam, *CNA* Clozapine, *CLP* Clonazepam, *CLNE* Clotiapine, *LZP* Lorazepam, *FNA* Phenytoin, *FBTL* Phenobarbital, *LVTA* Levetiracetam, *ARZ* Aripiprazole, *DPM* Diazepam, *BNA* Benzodiazepine, PZE Promazine, *CHZE* Chlorpromazine, *TPO* Topiramate, *ZA* Hydroxyzine, *PO* Valproate, *OLZA* Olanzapine, *kLP* Klonopin, *XINA* Dexmedetomidine, *TRCS* Tricyclics, *LZPM* Lorazepam, *GBPA* Gabapentin, *LTM* Lithium, *PDA* Pimozide, *CLO* Chlorprothixene, *TNO* Thioxanthene, *HPL* Haloperidol, *FTA* Phenothiazine, *SRT* Sertraline, *CL* Clomipramine. Indication of DBS. *RUA* Reduce Uncontrolled Aggressiveness, *TD* Tardive Dyskinesia

### Neurosurgical parameters

In all the articles reviewed (Table 3), surgical planning was performed under stereotactic guidance with pre and postoperative magnetic resonance imaging and computed tomography fused images with the aid of software for target point orientation. Except for 1 study [38], no surgical parameters were determined. In 47.06% of the publications [6, 12, 23–25, 31, 32, 41], the brain target was the posteromedial hypothalamic nuclei (pHypN), a well-known target; while other groups proposed earlier targets [5, 40] or ventral regions [30, 34, 36, 37, 39] or a combination of short-subcortical brain regions [35, 38, 40]. Similarly, in most studies, the technique and electrode implantation coordinates were reported, although, in 4 articles, they were not reported [30, 35, 38, 39]. Similarly, 8 studies described the brand, and model of the electrode used in surgical implantation (Medtronic 3389). 4 registries used another reference [5, 30, 33, 39] and 6 show no information in this regard [34–38, 40]. Finally, stimulation parameters were described in 15 articles (41 patients). Voltage intensity ranged between 0.5 V and 6.5 V, frequency between 15 and 185 Hz, and pulse width between 60 and 360 μs. In 3 studiesthis information was not recorded [24, 35, 37].

**Table 3:** Brain target and stimulation parameters

First autor, Year	Target	Implantation	Paramentes
Benedetti-Isaac, 2015	PHypN	Preop image MRI + CT. Method Franzini et al., 2008 [42]. Medtronic 3389	PTE1: 2,7V, 185Hz; 90μs; Pt2: 2,4 V, 185Hz; 90μs; PTE3: 2,8V, 185Hz, 90μs
Benedetti-Isaac, 2021	PHypN	Pre/postop image MRI + CT. Method Franzini et al., 2005, 2008 [42, 43]. Medtronic 3389	N/A
Benedetti-Isaac, 2023a	PHypN	Intraop Image MRI + CT. Method Franzini et al., 2005, 2013. Medtronic 3389	2V, 180Hz, 90μs, 180/s. With settings 3-5V per patient
Benedetti-Isaac, 2023b	PHypN	Preop image MRI + CT. Method Franzini et al., 2005, 2010, 2013 [12, 43]. Medtronic 3389	2V, 180Hz, 90μs
Escobar-Vidarte, 2022	PHypN	Preop image MRI + CT. Method Franzini et al., 2013 [12]. Medtronic 3389	1-3V, 180Hz, 60μs; With progressive settings 3V
Franzini, 2013	PHypN	Pre/post-op image. Method: 3 mm behind the MCP, 5 mm below this point, and 2 mm lateral from the midline. Medtronic 3389	1-3V, 185Hz, 60-90μs
Giordano, 2016	VC/VS	Pre/post-op image MRI + Method:10.56 (left VC/VS) and 10.15 (right VC/VS) anterior, 1.10 dorsal to the midcommissural line, and 7 mm (left VC/VS) and 10 mm (right VC/VS) lateral to the sagittal plane. 2 precoronal 14-mm diameter burr holes located 10 mm anterior to the coronal suture, and 30 mm lateral to the midline following an oblique trajectory. Medtronic 3391.	2.5 V, 130 Hz, 210μs
Harat, 2021	Nacc	Preop image MRI + CT. Method: 7 mm lateral to the intercommissural line and 4–5 mm ventral/2–3 mm anterior AC. N/A electrode type.	N/A
Heiden, 2022	Amigdala	Preop image MRI + CT + Rx. N/A coordinates of Implantation. Medtronic 3389/3387	6,5V, 130Hz, 90μs
Kakko, 2019	GPi, ALIC	Preop imag MRI. Medtronic N/A reference and coordinates of Implantation.	N/A
López Rios, 2022	PHypN	Pre/post-op image MRI + CT. Method: 3 mm posterior, 2 mm above the superior margin of the red nucleus, and 2 mm lateral to the lateral wall of the third ventricle, with reference to the MCP. Medtronic 3387	0.9–3.3V, 180Hz, 100μs 1–3.3V, 180Hz, 100μs
Maley, 2010	PH, OFB	Pre/post-op image MRI + CT. Method: 15 mm AMCP, 5 mm lateral to the MSP, and with contacts level to the plane connecting the AC-PC and traversing up to 3 mm ventral to AC-PC. Medtronic. N/A reference.	DBS initial: 4V, 130Hz, 120μs. 1Week: 2.8V, 55Hz, 270μs. 5 months: 2.8V, 40Hz, 360μs. 12 months: 2.5V, 20Hz, 360μs
Micieli, 2017	PH, VT, RN	Pre/post-op image MRI + CT. Method: 1 mm behind the MCD and 5 mm below the AC-PC and 2 mm lateral to the lateral wall of the third ventricle. N/A electrode	100 μA, 200Hz, 150μs
Park, 2017	Nacc	Pre/post-op image MRI + CT. Method: 2.5 mm rostral to the anterior border of AC, 6.5 mm lateral from the midline, and 4.5 mm ventral to AC.	3-5V, 130Hz, 90μs.
Stocco, 2014	GPi, ALIC	N/A	PTE1: Gpi bipolar: 3.3V, 80Hz, 120μs. PTE2: Monopolar GPi: 2.5-50Vr/2.0-5Vl; 40-120Hz, 120-210μs; Bipolar adjustment ALIC PTE2: 2.0-2.5V; 80-200Hz; 100-210μs.
Sturm, 2012	BA	Pre/post-op image MRI + CT. N/A coordinates of Implantation. Medtronic 3387	2-6.5V, 130Hz, 120μs.
Torres, 2013/2020	PHypN	Intraop Image MRI + CT. Method Franzini et al., 2005, 2008 [42, 43]. Medtronic 3389	2013: 1 Year: 0.5-0.7V; 15Hz; 330-350μs. Adjustments of 1.8V, 130Hz, 210μs
			2020: 15Hz, 450μs, with gradual increase.

Target. *pHypN* Posteromedial hypothalamic nuclei, *VC/VS* Anterior limb of the internal capsule, *Nacc* Nucleus accumbens, *GPi* Globus pallidus interna, *ALIC* Anterior limb of the internal capsule, *PH* Posteromedial Hipotalamus, *OFB* Orbitofrontal cortex, *VT* Ventral Thalamus, *RN* Red Nucleus, *BA* Basolateral amygdaloid. Implantation. *Preop* Preoperative, *Postop* Postoperative, *Intraop* Intraoperative, *MCP* Midcommissural point, *AC* Anterior Commissure, *AMCP* Anterior Midcommissural Plane, *MSP* Midsagittal Plane, *AC-PC* anterior commissural-posterior commissural line, *N/A* not available

### Clinical outcomes, effectiveness, and complications

The case series used a heterogeneous combination of instruments to assess different aspects of the patients. Therefore, it was necessary to restrict the analysis to tests that allowed the assessment of pre- and post-intervention aggressiveness, with a clinical follow-up of at least 12 months.

In this regard, as seen in Table 4, few studies reported the application of an objective scale to assess intellectual disability [5, 12, 31] and autism [23, 30, 36]. This is due to the severity of symptoms. Regarding aggressiveness, only 5 records used a specialized scale (OAS) [12, 23–25, 31], and 6 groups used a modified version (MOAS) [5, 32–34, 37, 40]. One of these studies did not show test scores [34], and another record assessed the risk of aggression (BPAQ) [37]. In contrast, 2 studies did not show data on assessments of aggression [35, 40]. On the other hand, only one group assessed adaptive functioning with a specialized scale (ICAP) [6, 41], and 4 groups assessed other items [30, 36, 38, 39].

**Table 4:** Clinical results

First autor, year	Clinical scales	Clinical outcomes (Pre-Post DBS)	Positive results	Clinical Improvement %	Effect size
Benedetti‐Isaac et al., 2015 [25]	OAS	PTE1:9/1; Pte2:11/0; PTE3: 8/8	2/3	PTE1: 88%, Pt2: 90%, PTE3: 0%	N/A
Benedetti‐Isaac et al., 2021 [24]	OAS	Pre-ME: 17,73 ± 2,18 vs control 6 mos: 9,68 ± 4,02, vs 12 mos: 5,94 ± 2,27, vs 18 mos: 5,1 ± 1,91	18/19	6,12,18 mos: 45.4%, 66,5%, 71,2%	*P:*0,01; *d* = 3,78
Benedetti‐Isaac, et al., 2023a [22]	OAS, ADOS-2, WISC-IV	Pre-ME:18,60 ± 2,07 vs 6 mos: 11,80 ± 4,87, vs 12 mos: 6.80 ± 1.32, vs 8 mos: 6.60 ± 3.13	5/5	6, 12, 18 mos: 36,5%, 63,4%, 64,5%	*P*:0,00; *d* = 4,81
Benedetti‐Isaac, et al., 2023b [23]	OAS, WISC-IV	Pre-ME: 18.42 ± 3.03 vs 6 mos: 8.92 ± 3.58, vs 12 mos: 5.64 ± 2.00, vs 18 mos: 4.57 ± 0.93	12/12	6, 12, 18 mos: 51,5%, 69,4%, 75.2%	*P*:0,01; *d* = 3.75
Escobar Vidarte et al., 2022 [32]	MOAS	27.62 vs 15,57	6/8	42,67%	*P*:0,02; *d* = 1.01
Franzini et al., 2013 [12]	OAS, IQ	10 vs 1,5	2/2	85%	N/A
Giordano et al., 2016 [5]	MOAS, CPM	MOAS Pre: 34. Pos: 7 y 11 CPM ME global: 45	1/1	67,7% y 79,5%	-
Harat et al., 2021 [37]	MOAS, BPAQ	PTE1 MOAS 60 vs 1. BPAQ: 117 vs 58 PTE2 MOAS: 11 vs 1. BPAQ: 102 vs 90	1/2	PTE1: 98.3%, 50,4%. PTE2: 90.9%, 11,7%	N/A
Heiden et al., 2022 [39]	ERBI	ERBI Initial: 110; 6-month check-up: 160	1/1	45%	N/A
Kakko et al., 2019 [35]	N/A	N/A	1	N/A	N/A
López Ríos et al., 2022 [33]	MOAS, EQ-5D-5L	MOAS Pre-Post DBS: 40/3	1/1	MOAS: 92,5%	-
Maley et al., 2010 [40]	N/A	N/A	1/1	N/A	N/A
Micieli et al., 2017 [34]	MOAS, QOLS	N/A	4 / 4	N/A	N/A
Park et al., 2017 [36]	GCI-S, GCI-I, ABC, CY-BOCS, K-ARS, SRS	CGI-S: pre: 6; W4: 5; Year2: 4 CGI-I: pre:(No); W1:4; W4: 3; Year2: 2 ABC: pre: 106. W4: 71; Year2: 40. Year2: 7	1/1	CGI-S: W4: 16,6%; Year2: 33,3% CGI-I: W4: 25%; Year2: 50% ABC: W4: 33%; Years2: 62,2%	
Stocco, et al., 2014 [38]	JHMRS	PTE1: Pre-DBS: 30; 13 mos: 0 PTE2: Pre-DBS: 50; 13 mos: 50	1/2	PTE1: 100%; PTE2: 0%	N/A
Sturm et al., 2012 [30]	CGI, ADI-R	CGI (HS: 20/20; CNV: 8/7; CERR: 18/13)	1/1	N/A	N/A
Torres et al., 2013, 2020 [6, 41]	ICAP, DSM-IV GAF, MBI	ICAP: -38,5 ± 5,3 vs -16,3 ± 4,7	1/1	57,14%	N/A
		ICAP: -38/-18		52%	N/A

Clinical scales. *OAS* Overt Aggression Scale, *MOAS* Modified Overt Aggression Scale, *BPAQ* Buss-Perry Aggression Questionnaire (Amity version), *ERBI* Early Rehabilitation Barthel Index, *EQ-5D-5L* European Quality of Life5 Dimensions, *CGI-S* The Clinical Global Impression-Severity, *GCI-I* Clinical Global Impairment-Impression, *ABC* Antecedent, Behavior, Consequence, *JHMRS* John’s Hopkins motor stereotypy rating scale, *CGI* Clinical Global Impression Severity Scale, *ICAP* Inventory for Client and Agency Planning. *CPM* Clinical outcomes, Coloured progressive matrices; ADOS-2 Observational Scale for the Diagnosis of Autism, *WISC-IV* Wechsler Intelligence Scale for Children, *ADI-R* interview for autism detection, *IQ* Intelligence Quotient Classification, *DSM-IV GAF* Global Assessment of Functioning, *QOLS* The Quality-of-Life Scale, *MBI* Maladaptive Behavior Index Spanish version (MBI of the ICAP). *P* t of Student, *d* effect size, *N/A* Not available

Thus, in 53/65 patients, objective scales were applied to measure aggressiveness pre- and post-DBS. However, changes in aggressiveness were reported in only 51 subjects, considering a minimum clinical follow-up of 12 months. Therefore, an overall clinical improvement of 94.2% was identified in 48/51 patients. However, the effect size could only be calculated in 4 studies with significant results [23, 24, 31] and with data available in the OAS (*n* = 37/51); identifying a considerable effect size in these registries, which ranged from 3.71 to 4.81. For MOAS [32] (*n* = 6/51), a significant effect (*d* = *1*.01) was found in a single study. Finally, the mean performances of patients assessed separately on the OAS and MOAS, pre and post-surgery, were pooled. An intra-group analysis was performed with Student’s t, and the overall effect size of DBS on the aggressiveness of pediatric patients was calculated. The result was a considerable effect size in both instruments (OAS: *d* = *4*.32; MOAS: *d* = *1*.46).

On the contrary, unfavorable results were observed in 16 patients (Table 5). It was due to ganglio-basal bleeding [1, 24]. Also infections were observed in the operative area or at the battery testing site in 3 patients, for which the device had to be explanted [32, 34, 37]. In 2 patients adverse effects were observed, leading the family members to desist from continuing with the procedure [6, 32, 41]. In 2 cases it was necessary to adjust the stimulation parameters, to obtain an optimal response [33], or to reduce adverse symptoms [34]. In 6 patients the pulse generator battery ran out [30, 32, 33, 37]. Due to it, the initial symptoms of aggressiveness returned. They were reduced or eliminated with the change of batteries of the device. In one of these subjects, it was due to the patient’s failure to comply with the clinical control [37]. In this sense, in 3 patients it was not possible to perform an adequate follow-up because aggressiveness returned to initial levels a couple of months after starting brain stimulation [25, 38] or due to non-compliance with medical controls [39].

**Table 5:** Complications

First autor, year	Patients	Description
Benedetti‐Isaac et al., 2015 [25]	2/3	PTE 1: Died due to causes other than deep brain stimulation after the last follow-up. PTE 3: After two months, aggressiveness returned to baseline.
Benedetti‐Isaac et al., 2021 [24]	1/19	1 patient with basal ganglia hemorrhage
Benedetti‐Isaac, et al., 2023a [22]	-	None
Benedetti‐Isaac, et al., 2023b [23]	-	None
Escobar Vidarte et al., 2022 [32]	5/8	During follow-up, patients 1, 2, and 5 experienced GPI issues and increased aggression, which improved after generator replacement and resumption of hypothalamic stimulation. PTE 10: Device replacement required due to surgical site infection. PTE 11: As the stimulation parameters were increased, conjugate lateral gaze deviation occurred. The family requested explantation.
Franzini et al., 2013 [25]	-	None
Giordano et al., 2016 [5]	-	None
Harat et al., 2021 [2]	2/2	PTE 1: He received the first DBS (Naac) with some improvement, he underwent a second intervention in the posteromedial thalamus, he suffered infection, and the device was removed. PTE 2: Malfunction of the Impulse Pulse Generator (IPG).
Heiden et al., 2022 [39]	1/1	No clinical follow-up occurred due to missed follow-up appointments.
Kakko et al., 2019 [35]	-	None
López Ríos et al., 2022 [33]	1/1	Parameters were adjusted several times to improve treatment efficacy because of persistent insomnia and agitation and to minimize the mild side effects associated with higher-voltage stimulation.
Maley et al., 2010 [40]	1/1	Several stimulation adjustments were necessary because of the aggression and other pathologies observed until 12 months of age; after changing the parameters, aggressiveness decreased.
Micieli et al., 2017 [34]	2/4	PTE 1: Battery site infection. PTE 2: Transient dystonia of the left upper extremity improves with changes in stimulation.
Park et al., 2017 [36]	-	None
Stocco, et al., 2014 [38]	1/2	PTE 2: stereotypes back to their original condition
Sturm et al., 2012 [30]	1/1	The battery discharged in the 10th month, leading to the return of aggression. Aggressiveness diminished after battery change.
Torres et al., 2013, 2020 [6, 41]	1/1	2013: None
		2020: Increase in blood pressure, heart rate, and headache.

### Analysis of study quality and bias

Finally, the adapted NOS scale was applied, with an average score of 4.1 points, with a range of 2.25 to 5 (Table 6). Applying the 5 criteria of the adapted NOS, 9 studies were identified with excellent ratings (4.75 to 5 points), 7 studies obtained a score of fair (3 to 3.75 points), and 2 studies in the poor category (2.25 to 2.50 points). Regarding the assessment of bias (Table 7), 9 studies show a low level of bias (11–13 points), 8 show a medium risk (5–10 points) and only one study shows a high risk of bias (1–4 points).

**Table 6:** Evaluation of the quality of the studies using the NOS-adjusted scale

Study	Criteria 1	Criteria 2	Criteria 3	Criteria 4	Criteria 5	Score	Quality
Benedetti‐Isaac et al., 2015 [25]	1	1	1	1	1	5,00	Excellent
Benedetti‐Isaac et al., 2021 [24]	1	1	1	0,75	1	4,75	Excellent
Benedetti-Isaac et al., 2023a [22]	1	1	1	1	1	5,00	Excellent
Benedetti‐Isaac et al., 2023b [23]	1	1	1	1	1	5,00	Excellent
Escobar Vidarte et al., 2022 [32]	1	1	1	1	1	5,00	Excellent
Franzini et al., 2013 [12]	1	1	1	1	1	5,00	Excellent
Giordano et al., 2016 [5]	1	1	1	1	1	5,00	Excellent
Harat et al., 2021 [37]	1	1	1	0,75	1	4,75	Excellent
Heiden et al., 2022 [39]	1	1	0	0,75	0	2,75	Poor
Kakko et al., 2019 [35]	1	1	0	0,25	0	2,25	Poor
López Ríos et al., 2022 [33]	1	1	1	1	1	5,00	Excellent
Maley et al., 2010 [40]	1	1	1	0,75	0	3,75	Regular
Micieli et al., 2017 [34]	1	1	1	0,75	0	3,75	Regular
Park et al., 2017 [36]	1	1	1	0,75	0	3,75	Regular
Stocco & Baizabal-Carvallo, 2014 [38]	1	1	0,50	0,50	0	3,00	Regular
Sturm et al., 2012 [30]	1	1	1	0,50	0	3,50	Regular
Torres et al., 2013 [6]	1	1	1	0,75	0	3,75	Regular
Torres et al., 2020 [41]	1	1	1	0,75	0	3,75	Regular

Interpretation: 1 to 2.9 points: Poor; 3 to 3.9 points: Regular; 4 to 4.5: Good; 4.6 to 5: Excellent

The quality of each article was assessed by our team using the Newcastle–Ottawa Scale. We reviewed 5 NOS criteria, considering the technical characteristics of the brain stimulation studies. The items were scored either as positive (1) or negative (0). However, for items 2 and 4, each indicator described in the statement had to be met to obtain the total score)

Criteria 1. Did the sample represent all patients treated at the medical center, or did the study include all patients treated in the period under study? (1 point)

Criteria 2. ¿A correct diagnosis was made (ASD/ID) (0.5 points) and proper identification of the clinical problem? That is, intractable, drug-resistant, or uncontrolled aggression (0.5 points)

Criteria 3. Was the postoperative follow-up period at least 12 months? (1 point)

Criteria 4. Were all-important data cited in the report? Specifically, was a thorough clinical evaluation conducted? (0.25 points), as well as a relevant psychometric assessment (0.25 points); together with information on the implantation (0.25 points) and brain stimulation parameters (0.25 points)

Criteria 5. Were the results obtained using specialized aggression scales? (1 point)

**Table 7:** Evaluation of bias risk calculation: High: 1 to 4 points. Medium: 5 to 10 points. Low: 11 to 13 points

ID Study	Selection		Comparability	Outcome	Adequacy of follow up	Total Score	Risk
	Representativeness of exposed cohort (⋆)	Participant selection (⋆)	(⋆⋆⋆⋆)	Assessment of outcome (⋆⋆⋆⋆⋆)	(⋆⋆)		
Benedetti‐Isaac, 2015	⋆	⋆	⋆⋆⋆⋆	⋆⋆⋆⋆⋆	⋆	12	Low
Benedetti‐Isaac, 2021	⋆	⋆	⋆⋆⋆⋆	⋆⋆⋆⋆	⋆	11	Low
Benedetti-Isaac, 2023a	⋆	⋆	⋆⋆⋆⋆	⋆⋆⋆⋆	⋆	11	Low
Benedetti-Isaac, 2023b	⋆	⋆	⋆⋆⋆⋆	⋆⋆⋆⋆	⋆⋆	12	Low
Escobar Vidarte, 2022	⋆	⋆	⋆⋆⋆⋆	⋆⋆⋆⋆⋆	⋆	12	Low
Franzini, 2013	⋆	⋆	⋆⋆⋆⋆	⋆⋆⋆⋆	⋆	11	Low
Giordano, 2016	⋆	⋆	⋆⋆⋆	⋆⋆⋆⋆	⋆⋆	11	Low
Harat, 2021	⋆	⋆	⋆⋆⋆⋆	⋆⋆⋆⋆⋆	⋆⋆	13	Low
Heiden, 2022	⋆	⋆	⋆⋆	⋆		5	Medium
Kakko, 2019	⋆	⋆				2	High
López Ríos, 2022	⋆	⋆	⋆⋆⋆⋆	⋆⋆⋆⋆⋆	⋆⋆	13	Low
Maley, 2010	⋆	⋆	⋆⋆⋆	⋆⋆⋆	⋆	9	Medium
Micieli, 2017	⋆	⋆	⋆⋆⋆	⋆⋆⋆	⋆	9	Medium
Park, 2017	⋆	⋆	⋆⋆⋆	⋆⋆	⋆⋆	9	Medium
Stocco, 2014	⋆	⋆	⋆⋆⋆	⋆⋆	⋆	8	Medium
Sturm, 2012	⋆	⋆	⋆⋆	⋆⋆	⋆	7	Medium
Torres, 2013	⋆	⋆	⋆⋆⋆	⋆⋆	⋆	8	Medium
Torres, 2020	⋆	⋆	⋆⋆⋆	⋆⋆⋆	⋆	9	Medium

## Discussion

In this case series, we analyzed 18 publications, including retrospective studies and clinical cases, which represent a small number of records compared to other interventional modalities for children and adolescents with ASD, ID, and severe aggression [28, 36, 44]. The fact is that DBS is an uncommon procedure in clinical practice and is rarely used in minors because it involves invasive surgery that is recommended only for refractory conditions [23, 45]. Therefore, it is understandable that few studies are reported in the medical literature. Furthermore, in our analyses, the combination of search factors and the application of strict inclusion criteria further reduced the sample size. This explains why, although 100 subjects were identified in all records of interest, only 65 pediatric patients were included in the final analysis.

In addition, DBS is a costly procedure [29, 46], and there are cultural differences in the approach to these types of patients, as well as ethical and legal aspects that vary between countries. Therefore, we found few records in North America, where economic or legal aspects are more restrictive. On the contrary, a significant number of publications were identified in South America, with a concentration of interventions performed in Colombia, where there are several experiences with the application of DBS in pHypN for intractable aggression in children and adolescents with epilepsy [25, 32, 34], ASD and ID [23, 24, 31, 33]. Europe is the second continent where DBS has been used the most. In Italy, the first group to successfully apply DBS in subjects with aggression [42] validated the currently best-known implantation parameters [12, 42]; since then, they have applied DBS in various clinical conditions, but with few studies in pediatric ASD and ID. Similarly, in Spain [6, 41]; besides DBS, they have developed other forms of neurosurgical intervention in childhood autism [26]. In other continents, only one experience has been described in Asia [38].

### Pre-surgical conditions

This leads us to analyze the conditions for proposing DBS in the target population. Several years before implantation, it is essential to have a clear diagnosis of autism and/or intellectual disability in children, together with an assessment of the severity of the disorder or the level of functional impairment [47]. This is clinically documented in most studies, although few records used objective and specialized tests [12, 23–25, 31, 32, 34, 37, 40]. This is due to the severity of the symptoms, which makes it difficult to apply traditional tests. Early diagnosis of ASD and ID allows for greater clinical and therapeutic clarity, although it remains a challenge to diagnose, especially ASD, given the complexity of neurological maturation in children, the training of professionals, and the scarcity of instruments with adequate clinical utility values [48–52].

Subsequently, a clinical study with several years of follow-up is necessary, as reported by most teams, except for one record that lacked this information [35].Clinical follow-up supports the implementation of treatment options (pharmacological, psychological, and educational) and allows analysis of the impact of interventions on patients’ symptoms [53, 54]. However, DBS may sometimes be recommended to improve the patient's quality of life due to a history of medical iatrogenesis or negative effects of previous treatments, as reported in two records [35, 39]. In any case, clinical follow-up requires a multidisciplinary approach that includes psychiatrists, neurologists, and neuropsychologists [55, 56] to assess symptom refractoriness, functional impairment, aggression, and symptom severity. The neurosurgeon then reviews the case and proposes the intervention [57, 58].

In addition, a medical committee should be convened, and strict inclusion criteria applied to select candidates for surgery [59]. However, it is not always clear from the records reviewed whether all the necessary specialists were involved. Typically, a psychiatric evaluation with a neurosurgical focus was performed due to the severity of symptoms [9, 59] or the presence of disabling comorbidities, such as epilepsy [12, 25, 32–35, 37], dystonia [35], and psychiatric disorders [37, 38, 40]. Although recent studies show an interdisciplinary assessment ( [23, 31, 32, 34], few teams included medical committees [6, 23–25, 31, 32, 37, 41] or applied strict inclusion criteria [32, 33, 37]. The definition of selection criteria is crucial for the application of DBS in pediatric populations, but the paucity of studies and the lack of robust data limit this process.

Another critical aspect of the indication for DBS is the etiology of the aggressive disorder. In most studies, the cause of uncontrolled aggressive behavior was unknown, despite the presence of multiple neurological and psychiatric comorbidities, suggesting underlying congenital and/or genetic conditions. We identified the use of DBS in 14 patients with treatment-resistant epilepsy [25, 33–35, 37, 41], 8 patients with motor tics associated with Tourette syndrome [37, 39], and 1 patient with a genetic disorder [34], all of whom also presented with autism and predominantly severe intellectual disability.

Two points need to be made about aggression. First, aggression is not a disorder, but a comorbidity associated with many psychiatric or neurological disorders, with a significant prevalence in patients with neurodevelopmental disorders [60, 61]. In ASD and severe ID with low adaptive functioning, uncontrolled aggressive behavior is common and can be as debilitating as the core symptoms of these disorders [62, 63]. Second, due to the severity of symptoms and comorbidities, identifying the etiology of aggressive behavior can be problematic [64]. This can lead to a lack of focus in the clinical assessment of aggression in favor of assessing other symptoms, as observed in several studies [6, 30, 36, 38, 39, 41].

### Assessment of aggression

Although the etiology may be uncertain or there are various comorbidities, it is necessary to assess aggressiveness in patients undergoing DBS using objective tests or scales [17]. In this series of cases, the lack of a gold standard for evaluating aggressiveness was highlighted.

The first clinical instrument used to assess aggressiveness was the Overt Aggression Scale (OAS) [65], recommended for quickly evaluating aggressive behavior in hospital settings [66]. This scale measures verbal, physical, self and other aggression [67]. However, in this series of cases, it was only used in five records [12, 23–25, 31].

Next is the modified version of the OAS (MOAS), which is more appropriate for outpatient settings, where most individuals with problematic aggressive behavior are found. It contains the same 4 subcomponents for aggression and adds 2 items to assess anger and global aggression. It has demonstrated adequate psychometric and clinical values [66, 68]. Different versions of this instrument have been developed to study aggression in patients with severe mental illness in institutional settings [27, 69–72], to evaluate the anti-aggressive efficacy of beta-adrenergic blockers [73], or in individuals with traumatic brain injury [74, 75]. In this review, the MOAS was used in only 5 studies [5, 32–34, 37].

Finally, the Buss-Perry Aggression Questionnaire (BPAQ) [76], a scale with 4 dimensions (physical aggression, verbal aggression, anger, and hostility) known for assessing aggression in adults, was used in only one study [37].

One phenomenon that caught our attention was that several groups did not assess aggression directly, even though persistent aggressive behavior was described in all the records. Instead, they focused on other aspects, using scales to assess stereotypies [38], symptom severity and treatment effectiveness, attention deficit and obsessive–compulsive symptoms [30, 36], rehabilitation levels [39], and adaptive functioning [6, 41]. To complicate matters further, two groups mentioned using the MOAS but did not present the results [34, 40] or the tests were administered by a family member [33], which affects the quality of the results due to lack of expert assessment and informant bias [77]. Finally, one study did not report the use of an objective scale [35]. This situation warrants analysis, as it highlights the lack of international consensus in neurosurgery on important methodological aspects, such as the use of objective tests to contrast neurosurgical outcomes and increase the evidence for DBS.

### Clinical follow-up, effectiveness, and complications

In the reviewed studies, clinical follow-up was achieved in 51 of 65 pediatric patients. Using the results of aggression scales (OAS, MOAS) as an objective comparison criterion before and after DBS, a 94.2% improvement in aggression symptoms was observed (48 out of 51 patients). Notably, 44 out of 48 successful DBS implantations were performed in Colombia, 37 of them in the same clinical center on the north coast [22, 24, 25, 31]. This team has refined the surgical technique and methodological aspects, improving the safety and efficacy of DBS in children and adolescents. The other 7 patients were successfully treated in the central part of the country, demonstrating high standards of quality and safety.

This contrasts with other records where clinical follow-up was less than 12 months [38, 39], information was not available [35], or objective measures were not used to describe symptom evolution [34, 40].

In terms of procedural complications, atypical situations were found in 14 out of 65 patients. Three subjects experienced surgical site infection [32], battery site infection [34], or postoperative bleeding [24]. Evidence suggests that the risk of complications is related to the institution where the surgery is performed and the phenotypic conditions of the patients [78, 79]. This situation can be mitigated by the implementation of intraoperative and postoperative clinical measures [80].

In addition, 4 patients required adjustment of stimulation parameters due to persistent symptoms [33, 40] or complications with aggression and new symptoms [34, 40]. In one case, medication was required to control vascular symptoms [41]. Adjustment of DBS parameters can vary based on clinical outcomes [81], which is highly beneficial for improving psychiatric disorders or controlling side effects [82]. However, inappropriate use of these parameters can be counterproductive [83], and in some cases DBS must be accompanied by medication until optimal results are achieved [33, 41].

Unfortunately, DBS was discontinued in 2 patients. In the first case, it was due to lateral conjugate gaze deviation during the increase of stimulation parameters [32], which led the family to withdraw from the treatment. This was due to high intensity parameters that exceeded the therapeutic threshold and affected the oculomotor nerve fibers [7], which, although not significant for health, caused discomfort to the family. In the second patient [38], the intervention was discontinued due to the ineffectiveness of DBS in reducing self-injurious stereotypies, which returned to baseline levels. In addition, 2 subjects experienced problems with the implanted pulse generator due to noncompliance or interruption of medical follow-up [37, 39].

Evidence suggests that in some severe patients, DBS may not yield favorable results due to complex brain function and associated neurological dysfunction [3, 84]. Similarly, device failure and infections at the battery site can exacerbate psychiatric symptoms, increase hospitalizations, and require electrode removal [84–88]. This situation occurs because weeks may elapse between the postoperative period and the start of stimulation, during which the patient, if not managed with coercive measures, may self-injure and compromise the efficacy of stimulation as described previously [30, 35, 36, 38, 39].

Finally, in 4 datasets (6 subjects), the battery of the pulse generator was depleted [30, 32, 33, 37], causing relapse in patients who returned to pre-DBS symptoms. However, once the battery was replaced and brain stimulation was resumed, symptoms were reduced, and patients stabilized. Although the natural wear and tear of the implanted device batteries can be anticipated, it remains a significant event [89]. This demonstrates that the change in patients’ aggressiveness is due to DBS and not a placebo effect, as previously described [5, 12, 25, 32, 33, 37, 40].

### Target brain area

**Fig. 2:**
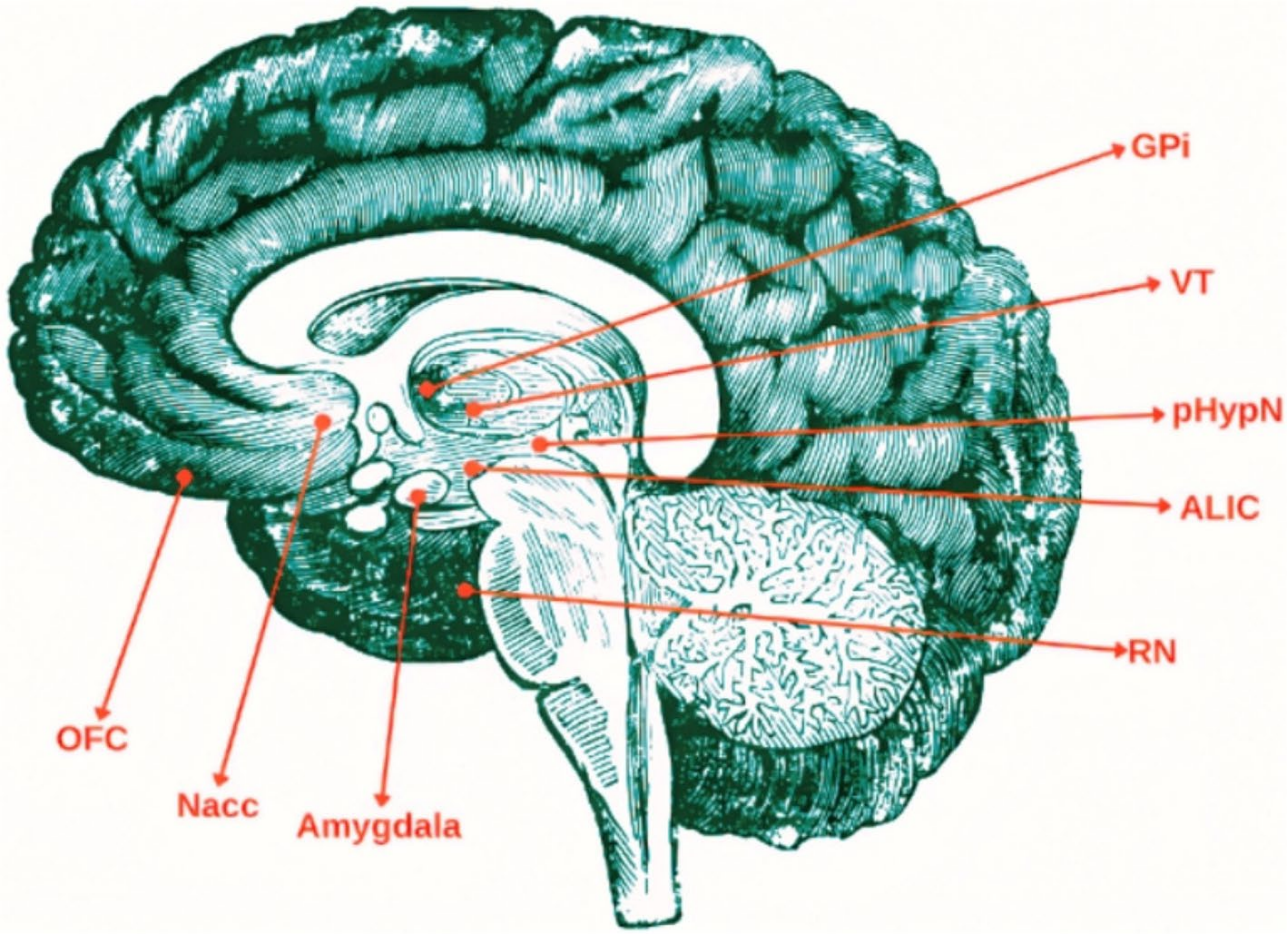
Synthesis of brain targets for DBS. OFC: Orbitofrontal cortex; Nacc: Nucleus accumbens; GPi: Globus palidus interna; VT: Ventral Thalamus; pHypN: Posteromedial hypothalamic nuclei; ALIC: Anterior limb of the internal capsule; RN: Red Nucleus

In this case series, a homogeneous methodology of surgical planning and reference to known electrodes was identified; however, there was no consensus on the target of brain stimulation. Below, we summarize the main brain targets for the treatment of aggression with DBS (Fig. 2).

In the 1960s, posteromedial hypothalamotomy and amygdalotomy emerged as treatment options for refractory pathological aggression [90, 91]. Both procedures showed significant improvements in symptom reduction; however, amygdalotomy had severe side effects, whereas hypothalamotomy had mild effects. In 1970, Sano’s group provided the first evidence that ablative lesioning of the pHyp could reduce aggressive and epileptic behavior, achieving clinical improvement in 95% of patients and delineating the “Sano triangle” in the posteromedial hypothalamus [92]. This work theorized connections between the pHyp, the amygdala, and the Papez circuit to explain disruptive aggressive behavior [93, 94]. The interconnections favored by the Papez circuit with the neocortex, limbic structures, and hypothalamus conceptualized this region as the anatomical substrate of central emotion and emotional experience [95].

Decades later, Franzini et al. [43] applied the first DBS for intractable aggressive behavior, targeting the posteromedial hypothalamus and detailing the implantation coordinates [12], defining the pHyp as the surgical target. Other groups have replicated this technique with similar results [6, 23–25, 31, 32, 41], including patients with autism and severe intellectual disability [25, 32, 41]. In this case series, most patients were treated according to Franzini’s implantation technique [44], although two recordings stimulated ventral regions with orbitofrontal projections connected to the pHyp [34, 40].

Besides the pHyp, the nucleus accumbens (NAcc) has also been a focus of interest for DBS implantation in patients with self-destructive and severely aggressive behaviors due to its role in the pathogenesis of neuropsychiatric disorders and its ability to induce locomotor activation, acting as an emotional-motor switch [96, 97]. In one study [37], the NAcc was selected after failure of DBS in the pHyp in two patients with OCD and autism, showing significant improvement in aggression and comorbidities, as previously described [4, 98]. However, the exact part of the NAcc that was stimulated was not clearly defined in Harat’s study [37]. In another study, the NAcc was selected for its role in the social reward system in ASD and a reduction in metabolism and cortical density in the prefrontal cortex was achieved [36].

The basolateral amygdala (BA) was another target in two datasets [30, 39], with historical evidence supporting its relevance [99, 100]. The BA, together with the centromedial and cortical nuclei, is part of the amygdaloid complex [101]. Stimulation of these nuclei, particularly the GABAergic and glutamatergic systems, has been implicated in autism [102, 103]. This has led to intriguing theoretical models linking the electrical disruption of amygdala circuits to clinical features of autism [104, 105].

The first study to analyze DBS in the basolateral amygdala (BA) for the treatment of pathological aggression was conducted by Sturm et al. [30] in a 13-year-old boy with ASD and intractable aggression. The choice of the BA was based on research linking it to the processing of emotions such as fear, anger, and socialization in autism [91, 105–111]. The results were positive, although without objective assessment, and it is possible that the improvement could be due to the activation of other brain regions [30]. On the other hand, Heiden et al. [39] applied DBS to a 10-year-old boy with ASD and severe mental retardation. Due to the lack of medical follow-up, sufficient information on the efficacy of DBS could not be obtained.

Finally, two studies reported interventions in the internal globus pallidus (GPi) and the anterior limb of the internal capsule (ALIC) in three patients with severe self-injurious involuntary movements [35, 38]. The first case involved a patient with ASD and moderate ID with intractable aggression and a long history of refractory antipsychotic medication, resulting in severe tardive dyskinesia and severe self-mutilation requiring hospitalization in the intensive care unit. At the age of 19, the patient received DBS in the GPi, which improved symptoms [35]. However, the study did not report stimulation parameters or objective clinical outcomes.

In the second case, DBS was applied to two patients aged 17 (GPi) and 19 (GPi + ALIC) with ASD, severe ID and severe self-injurious stereotypies. The target was chosen based on animal studies showing suppression of stereotypies by GPi stimulation [112]. Only one of the patients showed sustained improvement, while the other returned to pre-DBS status. It seems that DBS was used as a last therapeutic measure to provide relief from disabling stereotypies. DBS has shown variable results in GPi and ALIC [113, 114].

Given the diversity of brain stimulation targets, several key points stand out. First, to date, there is no single theoretical model that fully explains the interactions and functions of aggression in the brain [115–118]. Second, the precision of anatomical structures and the therapeutic effect of DBS are not entirely clear due to the complex neurophysiological connections involved [6, 36]. From a neurophysiological perspective, there are many connections from the posteromedial hypothalamus through the limbic system (including the amygdala and hippocampus) to the nucleus accumbens, as well as connections with the thalamus, motor cortex, and brainstem, which can explain the emotional and aggressive response.

Third, it is crucial to analyze symptoms and comorbidities in patients with ASD and severe ID when prescribing DBS [119, 120]. Different therapeutic targets have been used to treat different comorbidities associated with aggression, such as OCD, stereotypies, and social functioning problems, among others [14, 30, 34–39, 121]. However, the studies with the best outcomes and fewer complications selected the pHypN as the target.

Finally, the optimal stimulation parameters are uncertain [36], as therapeutic effects may take weeks to manifest, and the variability of pulse rates, frequencies, and voltages makes it difficult to establish reference values for DBS [41]. Applying high voltages or significantly increasing frequencies and pulse rates could cause injury or the emergence of other symptoms and disorders, as seen in several studies [33, 39]. Conversely, very low voltages would not stimulate effectively. Therefore, it seems that these parameters must be adjusted according to the characteristics and severity of the patient’s symptoms.

### Study limitations

There were several limitations to the records reviewed. First, the retrospective nature of the studies introduces observer bias, suggesting the need for controlled trials with blinded interdisciplinary evaluations to assess the efficacy of DBS in patients with intractable aggression. Second, it is crucial to establish a rigorous patient selection protocol that clearly details the steps and clinical decisions before neurosurgical intervention to avoid adverse effects.

A major limitation is the lack of a gold standard test to objectively assess aggression and adaptive functioning. While clinical observations are adequate, they should be supplemented with objective and specialized scales to better measure clinical improvement and the validity of DBS. In addition, it is necessary to record all relevant follow-up data for each patient, including test results and neurosurgical planning parameters, to add methodological value to the studies.

Finally, the meta-analysis was limited by incomplete information in the studies, such as demographic and clinical data, scores, and objective outcomes. Some studies mentioned the use of objective instruments without disclosing the results, which complicated the statistical analysis and the calculation of follow-up and clinical improvement. These difficulties highlight the need to strengthen methodological aspects in neurosurgical studies.

## Conclusions

In this case series, 100 subjects were analyzed, and 65 pediatric patients were identified. Of these, only 53 were assessed using objective scales to measure aggression, and 51 were clinically followed for at least 12 months. Of these, 48 showed a 94.2% clinical improvement in aggression indicators. DBS showed a significant positive effect, especially with the pHyp as the most effective surgical target, followed by the BA and, with less evidence, the GPi and ALIC.

Applying the quality criteria of the adapted NOS scale, only half of the studies (9/18) showed good therapeutic results in the target population. Conversely, 7 records were identified as fair and 2 as inadequate [35, 39]. The main problems identified in the studies were related to the lack of specialized scales to measure aggression (criterion 5), identified in 9 studies [6, 30, 34–36, 38–41], lack of data in the reports (criterion 4) in 4 records [30, 35, 38, 39], and clinical follow-up of less than 12 months (criterion 3) in 3 studies [35, 38, 39].

Results from the NOS scale present a less encouraging picture. However, DBS for intractable aggression in children and adolescents with ASD and severe ID can be safe and effective provided that rigorous and objective methodological parameters are followed. It is important to consider that the neuronal complexity of these patients, as well as neuroanatomical and neurophysiological changes during neurodevelopment, pose challenges for DBS [36, 47, 122]. Therefore, technical aspects such as the definition of implantation and electrical stimulation parameters, as well as the brain target, need to be addressed. A clear challenge that we believe would help increase the efficacy of DBS in children with ASD and ID is the application of strict criteria in patient selection, the involvement of professionals to perform pre-and post-intervention neuropsychological assessments, and the application of objective and specialized scales for aggression [123].

### Supplementary Information

Supplementary Material 1.

Supplementary Material 2.

## Data Availability

All data generated or analyzed during this study are included in this published article and its supplementary information files.

## References

[1] Liu A, Gong C, Wang B, Sun J, Jiang Z. Non-invasive brain stimulation for patient with autism: a systematic review and meta-analysis. Front Psychiatry. 2023;29:14. 10.3389/fpsyt.2023.1147327 PMC10338880 37457781

[2] Yan H, Elkaim LM, Venetucci Gouveia F, Huber JF, Germann J, Loh A, et al. Deep brain stimulation for extreme behaviors associated with autism spectrum disorder converges on a common pathway: a systematic review and connectomic analysis. J Neurosurg. 2022;137(3):699–708. 35061980 10.3171/2021.11.JNS21928

[3] O’Regan O, Doyle Y, Murray M, McCarthy VJC, Saab MM. Reducing challenging behaviours among children and adolescents with intellectual disabilities in community settings: a systematic review of interventions. Int J Dev Disabil. 2022;28:1–20. 10.1080/20473869.2022.2052416 PMC10916929 38456141

[4] Harat M, Rudaś M, Zieliński P, Birska J, Sokal P. Deep brain stimulation in pathological aggression. Stereotact Funct Neurosurg. 2015;93(5):310–5. 26227081 10.1159/000431373

[5] Giordano F, Cavallo M, Spacca B, Pallanti S, Tomaiuolo F, Pieraccini F, et al. Deep brain stimulation of the anterior limb of the internal capsule may be efficacious for explosive aggressive behaviour. Stereotact Funct Neurosurg. 2016;94(6):371–8. 27798944 10.1159/000449171

[6] Torres CV, Sola RG, Pastor J, Pedrosa M, Navas M, García-Navarrete E, et al. Long-term results of posteromedial hypothalamic deep brain stimulation for patients with resistant aggressiveness: clinical article. J Neurosurg. 2013;119(2):277–87. Available from: https://thejns.org/view/journals/j-neurosurg/119/2/article-p277.xml. Cited 2022 Aug 15. 23746102 10.3171/2013.4.JNS121639

[7] Gouveia FV, Germann J, Elias GJB, Hamani C, Fonoff ET, Martinez RCR. Case report: 5 Years follow-up on posterior hypothalamus deep brain stimulation for intractable aggressive behaviour associated with drug-resistant epilepsy. Brain Stimul. 2021;14(5):1201–4. 34371210 10.1016/j.brs.2021.07.062

[8] Adler BA, Wink LK, Early M, Shaffer R, Minshawi N, McDougle CJ, et al. Drug-refractory aggression, self-injurious behavior, and severe tantrums in autism spectrum disorders: a chart review study. Autism. 2015;19(1):102–6. 24571823 10.1177/1362361314524641

[9] Cleary DR, Ozpinar A, Raslan AM, Ko AL. Deep brain stimulation for psychiatric disorders: where we are now. Neurosurg Focus. 2015;38(6):E2. 26030702 10.3171/2015.3.FOCUS1546

[10] Davis RA, Winston H, Gault JM, Kern DS, Mikulich-Gilbertson SK, Abosch A. Deep brain stimulation for ocd in a patient with comorbidities: epilepsy, tics, autism, and major depressive disorder. J Neuropsychiatry Clin Neurosci. 2021;33(2):167–71 Available from: https://neuro.psychiatryonline.org/10.1176/appi.neuropsych.20060153. 33535803 10.1176/appi.neuropsych.20060153

[11] Doshi PK, Hegde A, Desai A. Nucleus accumbens deep brain stimulation for obsessive-compulsive disorder and aggression in an autistic patient: a case report and hypothesis of the role of nucleus accumbens in autism and comorbid symptoms. World Neurosurg. 2019;125:387–91. 30797934 10.1016/j.wneu.2019.02.021

[12] Franzini A, Broggi G, Cordella R, Dones I, Messina G. Deep-brain stimulation for aggressive and disruptive behavior. World Neurosurg. 2013;80(3–4):S29.e11-S29.e14. 22743202 10.1016/j.wneu.2012.06.038

[13] Bauerle L, Palmer C, Salazar CA, Larrew T, Kerns SE, Short EB, et al. Neurosurgery for psychiatric disorders: reviewing the past and charting the future. Neurosurg Focus. 2023;54(2):E8. Available from:https://thejns.org/focus/view/journals/neurosurg-focus/54/2/article-pE8.xml. Cited 2023 Aug 25. 36724525 10.3171/2022.11.FOCUS22622

[14] Gaitanis J. Deep brain stimulation for autism spectrum disorders. Neurosurg Focus. 2016;41(1):E12 Available from: https://thejns.org/focus/view/journals/neurosurg-focus/41/1/article-pE12.xml. Cited 2022 Oct 28. 27364254 10.3171/2016.1.FOCUS15603

[15] Storch EA, Cepeda SL, Lee E, Goodman SLV, Robinson AD, De Nadai AS, et al. Parental attitudes toward deep brain stimulation in adolescents with treatment-resistant conditions. J Child Adolesc Psychopharmacol. 2020;30(2):97–103. 31697591 10.1089/cap.2019.0134 PMC7047254

[16] Beszłej J, Wieczorek T, Kobyłko A, Piotrowski P, Siwicki D, Weiser A, et al. Deep brain stimulation: new possibilities for the treatment of mental disorders. Psychiatr Pol. 2019;53(4):789–806. 31760410 10.12740/PP/OnlineFirst/103090

[17] Ashkan K, Mirza AB, Tambirajoo K, Furlanetti L. Deep brain stimulation in the management of paediatric neuropsychiatric conditions: current evidence and future directions. Eur J Paediatr Neurol. 2021;33:146–58. 33092983 10.1016/j.ejpn.2020.09.004

[18] Page MJ, McKenzie JE, Bossuyt PM, Boutron I, Hoffmann TC, Mulrow CD, et al. The PRISMA 2020 statement: an updated guideline for reporting systematic reviews. BMJ. 2021;372:n71. Available from: https://www.bmj.com/lookup/doi/10.1136/bmj.n71. Cited 2023 Aug 23. 33782057 10.1136/bmj.n71 PMC8005924

[19] American Academy of Pediatrics. Ages & stages. Healthychilden.org; 2022. Available from: https://www.healthychildren.org/English/ages-stages/Pages/default.aspx. Cited 2023 Aug 23.

[20] Hardin AP, Hackell JM, Simon GR, Boudreau ADA, Baker CN, Barden GA, et al. Age limit of pediatrics. Pediatrics. 2017;140(3). Available from: https://publications.aap.org/pediatrics/article/140/3/e20172151/38333/Age-Limit-of-Pediatrics. Cited 2023 Aug 23. 10.1542/peds.2017-2151 28827380

[21] Thalheimer W, Cook S. How to calculate effect sizes from published research: a simplified methodology. Work Learn Res. 2002;9. Available from: www.work-learning.com. Cited 2022 Jul 17.

[22] Benedetti-Isaac J, Camargo L, Cardenas FP, López N. Effectiveness of deep brain stimulation in refractory and drug-resistant aggressiveness in autism spectrum disorder. Res Autism Spectr Disord. 2023;102: 102131. 10.1016/j.rasd.2023.102131

[23] Benedetti-Isaac J, Camargo L, Cardenas FP, López N. Effectiveness of deep brain stimulation in refractory and drug-resistant aggressiveness in autism spectrum disorder. Res Autism Spectr Disord. 2023;102:102131 Available from: https://linkinghub.elsevier.com/retrieve/pii/S1750946723000314. Cited 2023 Aug 23. 10.1016/j.rasd.2023.102131

[24] Benedetti-Isaac JC, Camargo L, Gargiulo P, López N. Deep brain stimulation in the posteromedial hypothalamic nuclei in refractory aggressiveness: post-surgical results of 19 cases. Int J Neuropsychopharmacol. 2021;24(12):977–8 Available from: https://academic.oup.com/ijnp/article/24/12/977/6358514. Cited 2022 Aug 15. 34448852 10.1093/ijnp/pyab059 PMC8653869

[25] Benedetti-Isaac JC, Torres-Zambrano M, Vargas-Toscano A, Perea-Castro E, Alcalá-Cerra G, Furlanetti LL, et al. Seizure frequency reduction after posteromedial hypothalamus deep brain stimulation in drug-resistant epilepsy associated with intractable aggressive behavior. Epilepsia. 2015;56(7):1152–61 https://onlinelibrary.wiley.com/doi/10.1111/epi.13025. Cited 2022 Oct 30. 26146753 10.1111/epi.13025

[26] Torres CV, Martínez N, Ríos-Lago M, Lara M, Alvarez-Linera J, Cabanyes J, et al. Surgery and radiosurgery in autism: a retrospective study in 10 patients. Stereotact Funct Neurosurg. 2021;99(6):474–83 Available from: https://www.karger.com/Article/FullText/516963. Cited 2022 Oct 28. 34474415 10.1159/000516963

[27] Margari F, Matarazzo R, Casacchia M, Roncone R, Dieci M, Safran S, et al. Italian validation of MOAS and NOSIE: a useful package for psychiatric assessment and monitoring of aggressive behaviours. Int J Methods Psychiatr Res. 2005;14(2):109–18. 16175880 10.1002/mpr.22 PMC6878297

[28] Seltzer MM, Krauss MW, Shattuck PT, Orsmond G, Swe A, Lord C. The symptoms of autism spectrum disorders in adolescence and adulthood. J Autism Dev Disord. 2003;33(6):565–81 Available from: https://link-springer-com.ezproxy.cuc.edu.co/article/10.1023/B:JADD.0000005995.02453.0b. Cited 2023 Aug 25. 14714927 10.1023/B:JADD.0000005995.02453.0b

[29] Goldberg DS. Justice, population health, and deep brain stimulation: the interplay of inequities and novel health technologies. 2012;3(1):16–20. https://doi-org.ezproxy.cuc.edu.co/101080/215077402011635626. Available from: https://www-tandfonline-com.ezproxy.cuc.edu.co/doi/abs/10.1080/21507740.2011.635626. Cited 2023 Aug 25.

[30] Sturm V, Fricke O, Bührle CP, Lenartz D, Maarouf M, Treuer H, et al. DBS in the baso-lateral Amygdala improves symptoms of autism and related self-injurious behavior: a case report and hypothesis on the pathogenesis of the disorder. Front Hum Neurosci. 2012;6:18459. 10.3389/fnhum.2012.00341 PMC3549527 23346052

[31] Benedetti-Isaac JC, Camargo L, Torres Zambrano M, Perea-Castro E, Castillo-Tamara E, Caldichoury N, et al. Deep brain stimulation may be a viable option for resistant to treatment aggression in children with intellectual disability. CNS Neurosci Ther. 2023;29(7):2010–7. Available from: https://onlinelibrary.wiley.com/doi/10.1111/cns.14156. Cited 2023 Aug 23. 36890650 10.1111/cns.14156 PMC10324351

[32] Escobar-Vidarte OA, Griswold DP, Orozco Mera J, Arango Uribe GJ, Salcedo JC. Deep brain stimulation for severe and intractable aggressive behavior. Stereotact Funct Neurosurg. 2022;100(4):210–3 Available from: https://www.karger.com/Article/FullText/521766. Cited 2023 Aug 23. 35100596 10.1159/000521766

[33] López Ríos AL, Germann J, Hutchison WD, Botero Posada LF, Ahunca Velasquez LF, Garcia Jimenez FA, et al. Long-term follow-up on bilateral posterior hypothalamic deep brain stimulation for treating refractory aggressive behavior in a patient with Cri du chat syndrome: analysis of clinical data, intraoperative microdialysis, and imaging connectomics. Stereotact Funct Neurosurg. 2022;100(5–6):275–81 Available from: https://www.karger.com/Article/FullText/526871. Cited 2023 Aug 23. 36446334 10.1159/000526871

[34] Micieli R, Rios ALL, Aguilar RP, Posada LFB, Hutchison WD. Single-unit analysis of the human posterior hypothalamus and red nucleus during deep brain stimulation for aggressivity. J Neurosurg. 2017;126(4):1158–64 Available from: https://thejns.org/view/journals/j-neurosurg/126/4/article-p1158.xml. Cited 2022 Aug 15 . 27341042 10.3171/2016.4.JNS141704

[35] Kakko K, Bjelogrlic-Laakso N, Pihlakoski L, Lehtimäki K, Järventausta K. Tardive dyskinesia should not be overlooked. 2019;29(1):72–4. Available from: https://home.liebertpub.com/cap, https://www.liebertpub.com/doi/10.1089/cap.2018.0084. Cited 2023 Aug 23. 10.1089/cap.2018.0084 30388034

[36] Park HR, Kim IH, Kang H, Lee DS, Kim BN, Kim DG, et al. Nucleus accumbens deep brain stimulation for a patient with self-injurious behavior and autism spectrum disorder: functional and structural changes of the brain: report of a case and review of literature. Acta Neurochir (Wien). 2017;159(1):137–43 Available from: https://link-springer-com.ezproxy.cuc.edu.co/article/10.1007/s00701-016-3002-2. Cited 2023 Aug 23. 27807672 10.1007/s00701-016-3002-2

[37] Harat M, Kiec M, Rudaś M, Birski M, Furtak J. Treating aggression and self-destructive behaviors by stimulating the nucleus accumbens: a case series. Front Neurol. 2021;11(12):706166. 10.3389/fneur.2021.706166 PMC8542713 34707553

[38] Stocco A, Baizabal-Carvallo JF. Deep brain stimulation for severe secondary stereotypies. Parkinsonism Relat Disord. 2014;20(9):1035–6 Available from: https://linkinghub.elsevier.com/retrieve/pii/S1353802014002399. Cited 2023 Aug 23. 25012696 10.1016/j.parkreldis.2014.06.019

[39] Heiden P, Weigel DT, Loução R, Hamisch C, Gündüz EM, Ruge MI, et al. Connectivity in deep brain stimulation for self-injurious behavior: multiple targets for a common network? Front Hum Neurosci. 2022;24(16):958247. 10.3389/fnhum.2022.958247 PMC9448926 36092644

[40] Maley JH, Alvernia JE, Valle EP, Richardson D. Deep brain stimulation of the orbitofrontal projections for the treatment of intermittent explosive disorder. Neurosurg Focus. 2010;29(2):E11 Available from: https://thejns.org/focus/view/journals/neurosurg-focus/29/2/2010.5.focus10102.xml. Cited 2023 Aug 23. 20672913 10.3171/2010.5.FOCUS10102

[41] Torres CV, Blasco G, García MN, Ezquiaga E, Pastor J, Vega-Zelaya L, et al. Deep brain stimulation for aggressiveness: long-term follow-up and tractography study of the stimulated brain areas. J Neurosurg. 2020;134(2):366–75 Available from: https://thejns.org/view/journals/j-neurosurg/134/2/article-p366.xml. Cited 2023 Aug 23. 32032944 10.3171/2019.11.JNS192608

[42] Franzini A, Messina G, Marras C, Villani F, Cordella R, Broggi G. Deep brain stimulation of two unconventional targets in refractory non-resectable epilepsy. Stereotact Funct Neurosurg. 2008;86(6):373–81. 19033706 10.1159/000175800

[43] Franzini A, Marras C, Ferroli P, Bugiani O, Broggi G. Stimulation of the posterior hypothalamus for medically intractable impulsive and violent behavior. Stereotact Funct Neurosurg. 2005;83(2–3):63–6. 15990469 10.1159/000086675

[44] Gouveia FV, Germann J, Elias GJ, Boutet A, Loh A, Lopez Rios AL, et al. Multi-centre analysis of networks and genes modulated by hypothalamic stimulation in patients with aggressive behaviours. Elife. 2023;12:e84566. 37212456 10.7554/eLife.84566 PMC10259501

[45] Manbeck C, Johnson T, Sharp G. A narrative review to guide treatment and care for children with Tourette syndrome. Brain Disord. 2023;11:100088 Available from: https://linkinghub.elsevier.com/retrieve/pii/S2666459323000252. Cited 2023 Aug 25. 10.1016/j.dscb.2023.100088 PMC12330826 40777575

[46] Pycroft L, Stein J, Aziz T. Deep brain stimulation: an overview of history, methods, and future developments. 2018;2:239821281881601. 10.1177/2398212818816017. Available from: https://journals.sagepub.com/doi/10.1177/2398212818816017. Cited 2023 Aug 25. 10.1177/2398212818816017 PMC7058209 32166163

[47] Graat I, Balke S, Prinssen J, de Koning P, Vulink N, Mocking R, et al. Effectiveness and safety of deep brain stimulation for patients with refractory obsessive compulsive disorder and comorbid autism spectrum disorder; a case series. J Affect Disord. 2022;299:492–7. 34952108 10.1016/j.jad.2021.12.089

[48] Daniels AM, Halladay AK, Shih A, Elder LM, Dawson G. Approaches to enhancing the early detection of autism spectrum disorders: a systematic review of the literature. J Am Acad Child Adolesc Psychiatry. 2014;53(2):141–52. 24472250 10.1016/j.jaac.2013.11.002

[49] Mozolic-Staunton B, Donelly M, Yoxall J, Barbaro J. Early detection for better outcomes: universal developmental surveillance for autism across health and early childhood education settings. Res Autism Spectr Disord. 2020;71:101496. 10.1016/j.rasd.2019.101496

[50] Guthrie W, Wallis K, Bennett A, Brooks E, Dudley J, Gerdes M, et al. Accuracy of autism screening in a large pediatric network. Pediatrics. 2019;144(4):e20183963. 31562252 10.1542/peds.2018-3963

[51] Robins DL, Casagrande K, Barton M, Chen CMA, Dumont-Mathieu T, Fein D. Validation of the modified checklist for autism in toddlers, revised with follow-up (M-CHAT-R/F). Pediatrics. 2014;133(1):37–45. 24366990 10.1542/peds.2013-1813 PMC3876182

[52] Choueiri R, Lindenbaum A, Ravi M, Robsky W, Flahive J, Garrison W. Improving early identification and access to diagnosis of autism spectrum disorder in toddlers in a culturally diverse community with the rapid interactive screening test for autism in toddlers. J Autism Dev Disord. 2021;51(11):3937–45. 33423215 10.1007/s10803-020-04851-3 PMC8510911

[53] Blasco García de Andoain G, Navas García M, González Aduna Ó, Bocos Portillo A, Ezquiaga Terrazas E, Ayuso-Mateos JL, et al. Posteromedial hypothalamic deep brain stimulation for refractory aggressiveness in a patient with weaver syndrome: clinical, technical report and operative video. Oper Neurosurg. 2021;21(3):165–71 Available from: https://journals.lww.com/10.1093/ons/opab149. 10.1093/ons/opab149 34017998

[54] Okun MS, Foote KD. Parkinson’s disease DBS: what, when, who and why? The time has come to tailor DBS targets. Expert Rev Neurother. 2010;10(12):1847–57. 21384698 10.1586/ern.10.156 PMC3076937

[55] Guinchat V, Cravero C, Lefèvre-Utile J, Cohen D. Multidisciplinary treatment plan for challenging behaviors in neurodevelopmental disorders. 2020. p. 301–21. Available from: https://linkinghub.elsevier.com/retrieve/pii/B9780444641489000223. 10.1016/B978-0-444-64148-9.00022-3 32977887

[56] Nuttin B, Wu H, Mayberg H, Hariz M, Gabriels L, Galert T, et al. Consensus on guidelines for stereotactic neurosurgery for psychiatric disorders. J Neurol Neurosurg Psychiatry. 2014;85(9):1003–8. 24444853 10.1136/jnnp-2013-306580 PMC4145431

[57] Deuschl G, Bain P. Deep brain stimulation for trauma: patient selection and evaluation. Mov Disord. 2002;17(S3):S102–11. 11948763 10.1002/mds.10150

[58] Smith AP, Bakay RAE. Frameless deep brain stimulation using intraoperative O-arm technology. J Neurosurg. 2011;115(2):301–9 Available from: https://thejns.org/view/journals/j-neurosurg/115/2/article-p301.xml. 21495822 10.3171/2011.3.JNS101642

[59] Rabins P, Appleby BS, Brandt J, DeLong MR, Dunn LB, Gabriëls L, et al. Scientific and ethical issues related to deep brain stimulation for disorders of mood, behavior, and thought. Arch Gen Psychiatry. 2009;66(9):931. 19736349 10.1001/archgenpsychiatry.2009.113 PMC2753479

[60] Cooper SA, Smiley E, Jackson A, Finlayson J, Allan L, Mantry D, et al. Adults with intellectual disabilities: prevalence, incidence and remission of aggressive behaviour and related factors. J Intellect Disabil Res. 2009;53(3):217–32. 19178617 10.1111/j.1365-2788.2008.01127.x

[61] Simó-Pinatella D, Mumbardó-Adam C, Alomar-Kurz E, Sugai G, Simonsen B. Prevalence of challenging behaviors exhibited by children with disabilities: mapping the literature. J Behav Educ. 2019;28(3):323–43. 10.1007/s10864-019-09326-9

[62] Matson J, Cervantes P. Assessing aggression in persons with autism spectrum disorders: an overview. Res Dev Disabil. 2014;35(12):3269–75 Available from: https://linkinghub.elsevier.com/retrieve/pii/S089142221400345X. 25178710 10.1016/j.ridd.2014.08.004

[63] Brown CE, Quetsch LB, Aloia LS, Kanne SM. Predictors of aggression, disruptive behavior, and anger dysregulation in youths with autism spectrum disorder. J Autism Dev Disord. 2023. Available from: https://link.springer.com/10.1007/s10803-022-05876-6. 10.1007/s10803-022-05876-6 36697931

[64] Matson J. Aggression and tantrums in children with autism: a review of behavioral treatments and maintaining variables. J Ment Health Res Intellect Disabil. 2009;2(3):169–87. 10.1080/19315860902725875

[65] Yudofsky S, Silver JM, Jackson W, Endicott J, Williams D. The overt aggression scale for the objective rating of verbal and physical aggression. Am J Psychiatry. 1986;143(1):35–9 Available from: http://psychiatryonline.org/doi/abs/10.1176/ajp.143.1.35. 3942284 10.1176/ajp.143.1.35

[66] Coccaro EF. The Overt Aggression Scale Modified (OAS-M) for clinical trials targeting impulsive aggression and intermittent explosive disorder: validity, reliability, and correlates. J Psychiatr Res. 2020;124:50–7. 32114032 10.1016/j.jpsychires.2020.01.007

[67] Silver J, Yudofsky S. The overt aggression scale: overview and guiding principles. J Neuropsychiatry Clin Neurosci. 1991;1(3):S22–9. 1821217

[68] Oliver PC, Crawford MJ, Rao B, Reece B, Tyrer P. Modified Overt Aggression Scale (MOAS) for people with intellectual disability and aggressive challenging behaviour: a reliability study. J Appl Res Intellect Disabil. 2007;20(4):368–72. 10.1111/j.1468-3148.2006.00346.x

[69] De Benedictis L, Dumais A, Stafford MC, Cote G, Lesage A. Factor analysis of the French version of the shorter 12-item Perception of Aggression Scale (POAS) and of a new modified version of the Overt Aggression Scale (MOAS). J Psychiatr Ment Health Nurs. 2012;19(10):875–80. 22295950 10.1111/j.1365-2850.2011.01870.x

[70] Foley SR, Browne S, Clarke M, Kinsella A, Larkin C, O’Callaghan E. Is violence at presentation by patients with first-episode psychosis associated with duration of untreated psychosis? Soc Psychiatry Psychiatr Epidemiol. 2007;42(8):606–10 Available from: http://link.springer.com/10.1007/s00127-007-0217-9. 17598060 10.1007/s00127-007-0217-9

[71] Nicholls TL, Brink J, Greaves C, Lussier P, Verdun-Jones S. Forensic psychiatric inpatients and aggression: an exploration of incidence, prevalence, severity, and interventions by gender. Int J Law Psychiatry. 2009;32(1):23–30. 19081629 10.1016/j.ijlp.2008.11.007

[72] Kay SR, Wolkenfeld F, Murrill LM. Profiles of aggression among psychiatric patients: I. Nature and prevalence. J Nerv Ment Dis. 1988;176(9). Available from: https://journals.lww.com/jonmd/fulltext/1988/09000/profiles_of_aggression_among_psychiatric_patients_.7.aspx. 10.1097/00005053-198809000-00007 3418327

[73] Knoedler DW. The modified overt aggression scale. Am J Psychiatry. 1989;146(8):1081b–1082 Available from: http://psychiatryonline.org/doi/abs/10.1176/ajp.146.8.1081b. 10.1176/ajp.146.8.1081b 2750991

[74] Giles GM, Mohr JD. Overview and inter-rater reliability of an incident-based rating scale for aggressive behaviour following traumatic brain injury: the Overt Aggression Scale-Modified for Neurorehabiltation-Extended (OAS-MNR-E). Brain Inj. 2007;21(5):505–11. 17522990 10.1080/02699050701311729

[75] Narevic E, Giles GM, Rajadhyax R, Managuelod E, Monis F, Diamond F. The effects of enhanced program review and staff training on the management of aggression among clients in a long-term neurobehavioral rehabilitation program. Aging Ment Health. 2011;15(1):103–12. 20924812 10.1080/13607863.2010.501070

[76] Buss AH, Perry M. The aggression questionnaire. J Pers Soc Psychol. 1992;63(3):452–9. 1403624 10.1037/0022-3514.63.3.452

[77] Kildahl AN, Oddli HW, Helverschou SB. Bias in assessment of co-occurring mental disorder in individuals with intellectual disabilities: theoretical perspectives and implications for clinical practice. J Intellect Disabil. 2023;28:174462952311541. 10.1177/17446295231154119 PMC11059834 36708367

[78] Tsuboi T, Cif L, Coubes P, Ostrem JL, Romero DA, Miyagi Y, et al. Secondary worsening following DYT1 dystonia deep brain stimulation: a multi-country cohort. Front Hum Neurosci. 2020;25:14. 10.3389/fnhum.2020.00242 PMC7330126 32670041

[79] Farrokhi F, Buchlak QD, Sikora M, Esmaili N, Marsans M, McLeod P, et al. Investigating risk factors and predicting complications in deep brain stimulation surgery with machine learning algorithms. World Neurosurg. 2020;134:e325–38. 31634625 10.1016/j.wneu.2019.10.063

[80] Sarica C, Iorio-Morin C, Aguirre-Padilla DH, Najjar A, Paff M, Fomenko A, et al. Implantable pulse generators for deep brain stimulation: challenges, complications, and strategies for practicality and longevity. Front Hum Neurosci. 2021;26:15. 10.3389/fnhum.2021.708481 PMC8427803 34512295

[81] Lozano AM, Lipsman N, Bergman H, Brown P, Chabardes S, Chang JW, et al. Deep brain stimulation: current challenges and future directions. Nat Rev Neurol. 2019;15(3):148–60. 30683913 10.1038/s41582-018-0128-2 PMC6397644

[82] Sellers KK, Cohen JL, Khambhati AN, Fan JM, Lee AM, Chang EF, et al. Closed-loop neurostimulation for the treatment of psychiatric disorders. Neuropsychopharmacology. 2024;49(1):163–78. Available from: https://www.nature.com/articles/s41386-023-01631-2. 10.1038/s41386-023-01631-2 PMC10700557 37369777

[83] Kuhn J, Gründler TOJ, Lenartz D, Sturm V, Klosterkötter J, Huff W. Deep brain stimulation for psychiatric disorders. Dtsch Arztebl Int. 2010;107(7): 105–13. Available from: https://www.aerzteblatt.de/int/archive/article/67821. 10.3238/arztebl.2010.0105 PMC2835924 20221269

[84] Vogt LM, Yan H, Santyr B, Breitbart S, Anderson M, Germann J, et al. Deep Brain Stimulation for Refractory Status Dystonicus in Children: Multicenter Case Series and Systematic Review. Ann Neurol. 2023;95(1):156–73. Available from: https://onlinelibrary.wiley.com/doi/10.1002/ana.26799. 10.1002/ana.26799 37714824

[85] Miron G, Strauss I, Fahoum F. De novo status epilepticus possibly related to battery depletion of anterior thalamic brain stimulator. Epileptic Disorders. 2022;24(1):151–5 Available from: https://onlinelibrary.wiley.com/doi/10.1684/epd.2021.1365. 34753709 10.1684/epd.2021.1365

[86] Miocinovic S, Ostrem JL, Okun MS, Bullinger KL, Riva-Posse P, Gross RE, et al. Recommendations for deep brain stimulation device management during a pandemic. J Parkinsons Dis. 2020;10(3):903–10. 32333552 10.3233/JPD-202072 PMC7458514

[87] Ooms P, Blankers M, Figee M, Mantione M, van den Munckhof P, Schuurman PR, et al. Rebound of affective symptoms following acute cessation of deep brain stimulation in obsessive-compulsive disorder. Brain Stimul. 2014;7(5):727–31. 25088461 10.1016/j.brs.2014.06.009

[88] Vora AK, Ward H, Foote KD, Goodman WK, Okun MS. Rebound symptoms following battery depletion in the NIH OCD DBS cohort: clinical and reimbursement issues. Brain Stimul. 2012;5(4):599–604. 22305344 10.1016/j.brs.2011.10.004

[89] Fakhar K, Hastings E, Butson CR, Foote KD, Zeilman P, Okun MS. Management of deep brain stimulator battery failure: battery estimators, charge density, and importance of clinical symptoms. PLoS One. 2013;8(3):e58665. 23536810 10.1371/journal.pone.0058665 PMC3594176

[90] Sano K, Yoshioka M, Ogashiwa M, Ishijima B, Ohye C. Postero-medial hypothalamotomy in the treatment of aggressive behaviors. Stereotact Funct Neurosurg. 1966;27(1–3):164–7. 10.1159/000103949 5955970

[91] Narabayashi H, Uno M. Long range results of stereotaxic amygdalotomy for behavior disorders. Stereotact Funct Neurosurg. 1966;27(1–3):168–71 Available from: https://www.karger.com/Article/FullText/103950. 10.1159/000103950 5334008

[92] Sano K, Mayanagi Y, Sekino H, Ogashiwa M, Ishijima B. Results of stimulation and destruction of the posterior hypothalamus in man. J Neurosurg. 1970;33(6):689–707. 5488801 10.3171/jns.1970.33.6.0689

[93] Tarnecki R, Mempel E, Fonberg E, Łagowska J. Some electrophysiological characteristics of the spontaneous activity of the amygdala and effect of hypothalamic stimulation on the amygdalar units responses. In: Stereotactic treatment of epilepsy. Vienna: Springer Vienna; 1976. p. 135–40. 10.1007/978-3-7091-8444-8_21 998337

[94] Hernando V, Pastor J, Pedrosa M, Peña E, Sola RG. Low-frequency bilateral hypothalamic stimulation for treatment of drug-resistant aggressiveness in a young man with mental retardation. Stereotact Funct Neurosurg. 2008;86(4):219–23. 18480600 10.1159/000131659

[95] Choi SH, Kim YB, Paek SH, Cho ZH. Papez circuit observed by in vivo human brain with 7.0T MRI super-resolution track density imaging and track tracing. Front Neuroanat. 2019;13:17. 30833891 10.3389/fnana.2019.00017 PMC6387901

[96] Heimer L, Van Hoesen GW, Trimble MR, Zahm DS. Anatomy of Neuropsychiatry: The new anatomy of the basal forebrain and its implications for neuropsychiatric illness. New York: Academic Press; 2008. p. 176.

[97] Mogenson G, Jones D, Yim C. From motivation to action: functional interface between the limbic system and the motor system. Prog Neurobiol. 1980;14(2–3):69–97 Available from: https://linkinghub.elsevier.com/retrieve/pii/0301008280900180. 6999537 10.1016/0301-0082(80)90018-0

[98] Tomycz ND, Whiting DM, Oh MY. Deep brain stimulation for obesity—from theoretical foundations to designing the first human pilot study. Neurosurg Rev. 2012;35(1):37–43. 21996938 10.1007/s10143-011-0359-9

[99] Langevin JP. The amygdala as a target for behavior surgery. Surg Neurol Int. 2012;3(2):40. 22826810 10.4103/2152-7806.91609 PMC3400485

[100] Narabayashi H, Mizutani T. Epileptic seizures and the stereotaxic amygdalotomy. Stereotact Funct Neurosurg. 1970;32(2–5):289–97 Available from: https://www.karger.com/Article/FullText/103429. 10.1159/000103429 4993814

[101] Yilmazer-Hanke DM, Faber-Zuschratter H, Blümcke I, Bickel M, Becker A, Mawrin C, et al. Axo-somatic inhibition of projection neurons in the lateral nucleus of amygdala in human temporal lobe epilepsy: an ultrastructural study. Exp Brain Res. 2007;177(3):384–99. 17006689 10.1007/s00221-006-0680-7

[102] Rubenstein JLR, Merzenich MM. Model of autism: increased ratio of excitation/inhibition in key neural systems. Genes Brain Behav. 2003;2(5):255–67. 14606691 10.1034/j.1601-183X.2003.00037.x PMC6748642

[103] State MW. The genetics of child psychiatric disorders: focus on autism and Tourette syndrome. Neuron. 2010;68(2):254–69. 20955933 10.1016/j.neuron.2010.10.004 PMC3292208

[104] Markram K, Markram H. The intense world theory – a unifying theory of the neurobiology of autism. Front Hum Neurosci. 2010;4:224. 21191475 10.3389/fnhum.2010.00224 PMC3010743

[105] Baron-Cohen S, Ring HA, Bullmore ET, Wheelwright S, Ashwin C, Williams SCR. The amygdala theory of autism. Neurosci Biobehav Rev. 2000;24(3):355–64. 10781695 10.1016/S0149-7634(00)00011-7

[106] Narabayashi H. From experiences of medial amygdalotomy on epileptics. 1980. p. 75–81. Available from: http://link.springer.com/10.1007/978-3-7091-8592-6_8. 10.1007/978-3-7091-8592-6_8 6937118

[107] Mempel E, Witkiewicz B, Stadnicki R, Łuczywek E, Kuciński L, Pawłowski G, et al. The effect of medial amygdalotomy and anterior hippocampotomy on behavior and seizures in epileptic patients. 1980. p. 161–7. Available from: http://link.springer.com/10.1007/978-3-7091-8592-6_20. 10.1007/978-3-7091-8592-6_20 6162367

[108] Narabayashi H, Nagao T, Saito Y, Yoshida M, Nagahata M. Stereotaxic amygdalotomy for behavior disorders. Arch Neurol. 1963;9(1):1–16 Available from: http://archneur.jamanetwork.com/article.aspx?articleid=564489. 13937583 10.1001/archneur.1963.00460070011001

[109] LeDoux J. The emotional brain, fear, and the amygdala. Cell Mol Neurobiol. 2003;23(4/5):727–38. 14514027 10.1023/A:1025048802629 PMC11530156

[110] Bauman M, Machado C, Amaral D, Schumann C. The Social Brain, Amygdala, and Autism. In: Understanding Autism. CRC Press; 2006. p. 227–53. Available from: http://www.crcnetbase.com/doi/10.1201/9781420004205.ch11.

[111] Santos A, Mier D, Kirsch P, Meyer-Lindenberg A. Evidence for a general face salience signal in human amygdala. Neuroimage. 2011;54(4):3111–6. 21081170 10.1016/j.neuroimage.2010.11.024

[112] Baup N, Grabli D, Karachi C, Mounayar S, François C, Yelnik J, et al. High-frequency stimulation of the anterior subthalamic nucleus reduces stereotyped behaviors in primates. J Neurosci. 2008;28(35):8785–8. 18753380 10.1523/JNEUROSCI.2384-08.2008 PMC6670823

[113] Akbarian-Tefaghi L, Akram H, Johansson J, Zrinzo L, Kefalopoulou Z, Limousin P, et al. Refining the deep brain stimulation target within the limbic globus pallidus internus for Tourette syndrome. Stereotact Funct Neurosurg. 2017;95(4):251–8. 28787721 10.1159/000478273

[114] Coubes P, Saleh C, Gonzalez V, Cif L. Deep brain stimulation of the globus pallidus internus and Gilles de la Tourette syndrome: toward multiple networks modulation. Surg Neurol Int. 2012;3(3):127. 22826816 10.4103/2152-7806.95424 PMC3400493

[115] Matthies S, Rüsch N, Weber M, Lieb K, Philipsen A, Tuescher O, et al. Small amygdala – high aggression? The role of the amygdala in modulating aggression in healthy subjects. World J Biol Psychiatry. 2012;13(1):75–81. 22256828 10.3109/15622975.2010.541282

[116] Rosell DR, Siever LJ. The neurobiology of aggression and violence. CNS Spectr. 2015;20(3):254–79. 25936249 10.1017/S109285291500019X

[117] Takahashi A, Miczek KA. Neurogenetics of aggressive behavior: studies in rodents. Curr Top Behav Neurosci. 2015;17:3–44 Available from: https://link.springer.com/chapter/10.1007/7854_2013_263. Cited 2022 Oct 28. 10.1007/7854_2013_263 PMC4092042 24318936

[118] Emberti Gialloreti L, Curatolo P. Autism spectrum disorder: why do we know so little? Front Neurol. 2018;17:9. 10.3389/fneur.2018.00670 PMC6107753 30174643

[119] Graat I, Figee M, Denys D. The application of deep brain stimulation in the treatment of psychiatric disorders. Int Rev Psychiatry. 2017;29(2):178–90. 28523977 10.1080/09540261.2017.1282439

[120] Marotta R. New therapeutic option in severe autism spectrum disorders: the deep brain stimulation in mesolimbic and mesocortical pathways. Acta Med Mediterr. 2020;36(3):1901–3.

[121] Segar DJ, Chodakiewitz YG, Torabi R, Cosgrove GR. Deep brain stimulation for the obsessive-compulsive and Tourette-like symptoms of Kleefstra syndrome. Neurosurg Focus. 2015;38(6):E12. 26030700 10.3171/2015.3.FOCUS1528

[122] Finisguerra A, Borgatti R, Urgesi C. Non-invasive brain stimulation for the rehabilitation of children and adolescents with neurodevelopmental disorders: a systematic review. Front Psychol. 2019;10(FEB):135. 30787895 10.3389/fpsyg.2019.00135 PMC6373438

[123] Salanova V, Witt T, Worth R, Henry TR, Gross RE, Nazzaro JM, et al. Long-term efficacy and safety of thalamic stimulation for drug-resistant partial epilepsy. Neurology. 2015;84(10):1017–25. 25663221 10.1212/WNL.0000000000001334 PMC4352097

