# Supplementary material for: Effectiveness of deep brain stimulation on refractory aggression in pediatric patients with autism and severe intellectual disability: meta-analytic review
Source: BMC Pediatr. 2024 Jul 30;24:487. doi: 10.1186/s12887-024-04920-x (PMC11290060; doi:10.1186/s12887-024-04920-x)
Supplement: Supplementary file 1 — Supplementary Material 1. [file 12887_2024_4920_MOESM1_ESM.docx]

**Appendix A.** NOS assessment guideline for bias in studies

| **Categories** | **Items** |
| --- | --- |
| **Selection** | |
| Representativeness of exposed cohort (⋆)  Points: a | a) It describes all eligible cases with an outcome of interest, from the same clinical or hospital center and is an appropriate sample of cases.  b) It is a group of selected users who do not belong to the medical center.  c) It is a group of voluntary patients from the community  d) There is insufficient information on the patient cohort. |
| Selection of participants (⋆)  Points: a | a) Subjects were included based on clinical criteria, interdisciplinary assessments, caregiver input, and clinical meetings?  b) Subjects were included based on clinical self-reports.  c) Incomplete information or unclear process?  d) No information or no description of the selection process. |
| **Comparability (**⋆⋆⋆⋆**)** | |
| (Based on methodological aspects)  Points = a, b, c y d | a) Clinical assessment of aggressive behavior  b) Psychometric assessment of aggressive behavior  c) Is a history of intractable aggressive behavior and exhaustion of traditional therapeutic procedures reported?  d) Was a medical board performed to select cases as candidates for neurosurgery?  e) Were partial aspects of the patients and the procedure reported?  f) Were other clinical issues evaluated?  No relevant information was found |
| **Outcome** | |
| Assessment of outcome (⋆⋆⋆⋆⋆)  Points: a, b, c, d y e | a) Objective scales were applied to assess pre and post DBS aggressiveness.  b) Planning parameters were reported  c) Reported surgical implantation  d) Reported average clinical improvement  e) Reported side effects, surgical corrections, adjustments and/or explantations.  f) Outcome description was done in a clinical manner.  g) Self-reports were used, based on descriptions made by caregivers.  h) No description |
| **Adequacy of follow up (⋆⋆)** | |
| Points: a y b | a) A postoperative clinical follow-up of 12 months or more was performed.  b) Pre-operative clinical follow-up equal to or greater than 12 months.  c) Post DBS clinical follow-up of less than 12 months  d) Pre DBS clinical follow-up of less than 12 months  e) No follow-up information |
