# Supplementary material for: Effectiveness of deep brain stimulation on refractory aggression in pediatric patients with autism and severe intellectual disability: meta-analytic review
Source: BMC Pediatr. 2024 Jul 30;24:487. doi: 10.1186/s12887-024-04920-x (PMC11290060; doi:10.1186/s12887-024-04920-x)
Supplement: Supplementary file 2 — Supplementary Material 2. [file 12887_2024_4920_MOESM2_ESM.docx]

**Appendix B. Characteristics of Excluded studies**

| Author-year | Cause to exclude |
| --- | --- |
| Dalic et al., 2022 | Follow-up study without surgical data to validate instruments. |
| Franzini et al., 2005 | Case study, participants older than pediatric age, ≤21 years. |
| Hernández Salazar et al., 2018 | Case study, the surgical technique utilized is not consistent with DBS. |
| Gouveia et al., 2023 | Retrospective connectivity study, compiling data from other publications. |
| Yan et al., 2022 | Pilot study, no case studies reported. |
| Salanova et al., 2015 | Randomized cohort study with long-term follow-up that excludes surgical data. |
| Torres et al., 2021 | Retrospective study result, the methodology used does not correspond to DBS. |
| Warsi et al., 2023 | Subjects had a primary epilepsy diagnosis and met criteria for refractory aggressiveness. |
